# Ginsenoside Rg1 Regulates Immune Microenvironment and Neurological Recovery After Spinal Cord Injury Through MYCBP2 Delivery via Neuronal Cell‐Derived Extracellular Vesicles

**DOI:** 10.1002/advs.202402114

**Published:** 2024-06-19

**Authors:** Yuluo Rong, Jiaxing Wang, Tao Hu, Zhongming Shi, Chuandong Lang, Wei Liu, Weihua Cai, Yongjin Sun, Feng Zhang, Wenzhi Zhang

**Affiliations:** ^1^ Department of orthopaedics Centre for Leading Medicine and Advanced Technologies of IHM The First Affiliated Hospital of USTC Division of Life Sciences and Medicine University of Science and Technology of China Hefei Anhui 230001 China; ^2^ National Center for Translational Medicine (Shanghai) SHU Branch Shanghai University Shanghai 200444 China; ^3^ Department of Orthopedics The First Affiliated Hospital of Nanjing Medical University Nanjing Jiangsu 210029 China; ^4^ Department of Orthopedics Second Affiliated Hospital of Naval Medical University Shanghai 200003 China

**Keywords:** extracellular vesicles, ginsenoside Rg1, microglial polarization, MYCBP2/S100A9 axis, oxidative stress, spinal cord injury

## Abstract

Spinal cord injury (SCI) is a severe neurological condition that frequently leads to significant sensory, motor, and autonomic dysfunction. This study sought to delineate the potential mechanistic underpinnings of extracellular vesicles (EVs) derived from ginsenoside Rg1‐pretreated neuronal cells (Rg1‐EVs) in ameliorating SCI. These results demonstrated that treatment with Rg1‐EVs substantially improved motor function in spinal cord‐injured mice. Rg1‐EVs enhance microglial polarization toward the M2 phenotype and repressed oxidative stress, thereby altering immune responses and decreasing inflammatory cytokine secretion. Moreover, Rg1‐EVs substantially diminish reactive oxygen species accumulation and enhanced neural tissue repair by regulating mitochondrial function. Proteomic profiling highlighted a significant enrichment of MYCBP2 in Rg1‐EVs, and functional assays confirmed that MYCBP2 knockdown counteracted the beneficial effects of Rg1‐EVs in vitro and in vivo. Mechanistically, MYCBP2 is implicated in the ubiquitination and degradation of S100A9, thereby promoting microglial M2‐phenotype polarization and reducing oxidative stress. Overall, these findings substantiated the pivotal role of Rg1‐EVs in neuronal protection and functional recovery following SCI through MYCBP2‐mediated ubiquitination of S100A9. This research offers novel mechanistic insights into therapeutic strategies against SCI and supports the clinical potential of Rg1‐EVs.

## Graphical abstract

The study reveals the potential mechanism by which extracellular vesicles (EVs) derived from ginsenoside Rg1‐pretreated neuronal cells (Rg1‐EVs) promote functional recovery after spinal cord injury. These findings demonstrate that Rg1‐EVs enhance motor function recovery in mice by enhancing microglia's polarization toward the M2 phenotype and reducing oxidative stress, driven by MYCBP2‐mediated ubiquitination and degradation of S100A9.

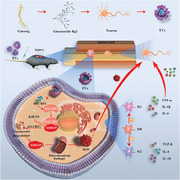

## 1 Introduction

The treatment and rehabilitation of spinal cord injury (SCI), which leads to significant neurological disability, is a critical area of focus in the field of neuroscience.^[^ ^1^ ^]^ Despite advances in therapeutic interventions, the existing approaches typically offer only partial alleviation of symptoms and associated complications, with full recovery of function remaining challenging. Following structural damage from the primary trauma, the injury site undergoes multiple secondary complications, such as severe inflammatory responses, oxidative stress (OS), and subsequent neuronal death.^[^ ^2^ ^]^ Persistent inflammation after SCI exacerbates these conditions, leading to prolonged recovery periods and suboptimal outcomes. Therefore, addressing the secondary inflammatory response is imperative for facilitating neural repair and improving functional outcomes.

Microglia, the resident macrophages of the central nervous system (CNS), assume a pivotal role in modulating neuroinflammation following SCI.^[^ ^3^ ^]^ Microglia can be polarized to M1 (proinflammatory) and M2 (anti‐inflammatory) phenotypes.^[^ ^4^, ^5^ ^]^ Following SCI, M1‐phenotype microglia contribute to the worsening of the inflammatory responsess by secreting proinflammatory factors, including tumor necrosis factor‐α (TNF‐α) and interleukin (IL)−1β. Additionally, the elevated permeability of the blood‐spinal cord barrier facilitates M1 microglia in releasing chemokines, further recruiting and enabling the infiltration of peripheral leukocytes. Such activities perpetuate the inflammatory cascade and accelerate excitotoxicity‐induced neuronal death.^[^ ^6^ ^]^ In contrast, M2‐phenotype microglia produce anti‐inflammatory cytokines, including IL‐10 and transforming growth factor‐β (TGF‐β), which constrain inflammatory reactions and intensify neuroprotection and neural tissue repair.^[^ ^7^ ^]^ Targeted modulation of microglial polarization encourages the transition from M1 to M2 phenotype through pharmacological, biological, or other alternative strategies, mitigates inflammatory damage, and augments nerve regeneration following SCI.

Recent research has increasingly focused on extracellular vesicles (EVs) as a promising therapeutic tool. These nano‐sized vesicles, released by various cell types under physiological and pathological conditions, encapsulate crucial information molecules that can modulate immune responses and boost cellular repair and regeneration. Notably, EVs derived from neuronal cells are critical to processes of neuroprotection and the repair and regeneration of neural tissues.^[^ ^8^ ^]^ Our previous research demonstrated that EVs secreted by M2‐phenotype macrophages carrying miR‐421‐3p inhibited the mammalian target of the rapamycin (mTOR) autophagy pathway, thereby reducing neuronal apoptosis and enhancing functional recovery following SCI.^[^ ^9^ ^]^ Moreover, evidence suggests that neuronal cell‐derived EVs can also support the repair of SCI by transporting miRNAs.^[^ ^10^, ^11^ ^]^ These studies highlight the significant role of EVs in facilitating neuronal communication and their potential therapeutic implications in the pathophysiological context of SCI repair.

The strategic enhancement of cell engraftment through pretreatment with cytokines, drugs, hypoxia, or physical stimuli is crucial for improving the biological functions of cells and strengthening paracrine signaling.^[^ ^12^, ^13^ ^]^ Ginsenoside Rg1, a primary active ingredient extracted from ginseng, has been extensively studied for its wide‐ranging pharmacological actions in various diseases. In neurological disorders, Rg1 is noted for its neuroprotective, neuroreparative, antioxidant, and anti‐inflammatory properties.^[^ ^14^ ^]^ For neurodegenerative diseases such as stroke, Parkinson's disease, and Alzheimer's disease, Rg1 has been shown to protect neuronal cells from damage, promote their survival, and facilitate functional recovery by reducing OS and the accumulation of neurotoxic substances.^[^ ^15^ ^]^ In the context of SCI, Rg1 inhibits inflammatory responses and OS, while also supporting neuronal growth and synapse formation, thus contributing to the reconstruction of neural networks and functional recovery.^[^ ^16^, ^17^ ^]^ Nonetheless, the impact of EVs derived from Rg1‐pretreated neuronal cells (Rg1‐EVs) on motor recovery after SCI has not been fully characterized. Furthermore, it remains to be clarified whether the observed therapeutic benefits are directly attributable to the genetic contents transported by these EVs.

**Scheme 1:**
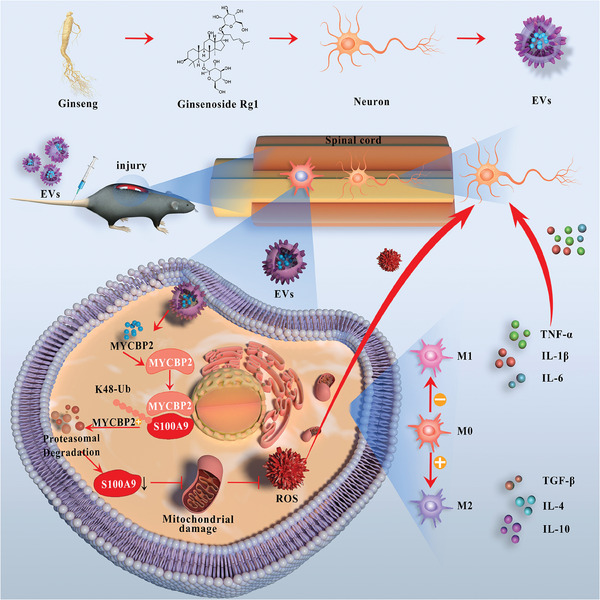
Schematic diagram of Rg1‐EVs regulating the immune microenvironment and neurological recovery after spinal cord injury.

This study demonstrated that MYCBP2, an E3 ubiquitin ligase, was notably enriched in Rg1‐EVs compared to untreated neuronal cells. The delivery of MYCBP2 via Rg1‐EVs facilitated the polarization of microglia toward the M2 phenotype, which in turn augmented functional motor recovery following SCI. Mechanistically, MYCBP2‐containing EVs modulated microglial polarization and reduced OS through the ubiquitin‐mediated degradation of S100A9 (**Scheme** **1**). This study reveals a potential mechanism through which Rg1‐EVs contribute to SCI treatment and provides new avenues for enhancing overall recovery and quality of life in affected patients.

## 2 Results

### 2.1 Identification of Rg1‐EVs

Extracellular vesicles were separated from the conditioned media of Rg1‐treated and untreated neuronal cells via ultracentrifugation. These vesicles were characterized using transmission electron microscopy (TEM), nanoparticle tracking analysis (NTA), and Western blot. TEM images illustrated that EVs and Rg1‐EVs exhibited similar cup‐like or spherical shapes (Figure S1A, Supporting Information). NTA results confirmed that EVs and Rg1‐EVs had comparable size distributions, with an average diameter of ≈120 nm (Figure S1B,C, Supporting Information). Notably, Rg1 treatment led to an increased release of EVs (Figure S1D, Supporting Information). The surface zeta potentials of EVs and Rg1‐EVs displayed no significant variances (Figure S1E,F, Supporting Information). Western blot assay unveiled the enrichment of key vesicular proteins, including CD9, CD63, CD81, ALIX, and TSG101 in EVs and Rg1‐EVs, whereas the endoplasmic reticulum protein calnexin was not expressed in EVs and Rg1‐EVs (Figure S1G, Supporting Information). Additionally, EVs were labeled with PKH26, cocultured with microglia, and observed under bright‐field and fluorescence microscopes (Figure S1H, Supporting Information). Confocal microscopic images indicated progressive uptake of EVs into microglia, as evidenced by increasing intracellular vesicle counts over the coculture period (Figure S1I,J, Supporting Information).

### 2.2 Rg1‐EVs Augment the Functional Recovery in SCI Mice

**Figure 1:**
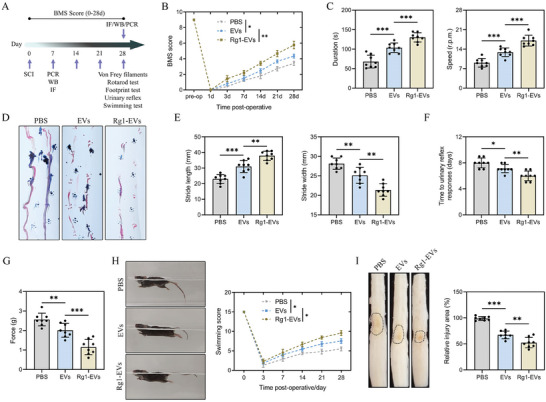
Rg1‐EVs facilitate functional recovery in SCI mice. A) Schematic representation of the in vivo experimental setup; B) Measurement of the BMS scores of mice receiving PBS, EVs, and Rg1‐EVs during the 28‐day recovery period post‐injury (*n* = 8); C) Statistical analysis of the results of rotarod test for the three groups (*n* = 8); D–E) Footprint analyses of the three groups (*n* = 8); F) Analysis of the recovery time of bladder reflexes in the three groups (*n* = 8); G) Statistical analysis of the results of the VFF test in the three groups (*n* = 8); H) Determination of LSS scores showing the function grade of mice 28 days post‐injury (*n* = 8); I) Statistical analysis of the gross morphology of the spinal cord and the injury site (*n* = 8); ^*^ *p <* 0.05; ^**^ *p <* 0.01; ^***^ *p <* 0.001.

Existing research has provided substantial evidence supporting that neuronal cell‐derived EVs contribute to the enhancement of functional recovery in mice following SCI.^[^ ^10^ ^]^ This current study explored the possibility of Rg1‐EVs in augmenting this recovery process. To test this hypothesis, we conducted a series of functional behavioral assessments over a 28‐day post‐SCI period, as delineated in **Figure** **1**A. The results indicated that mice receiving Rg1‐EVs, relative to the control EVs‐treated counterparts, exhibited markedly improved outcomes in walking capacity, hind paw positioning, and overall hindlimb locomotion, as evidenced by higher Basso Mouse Scale (BMS) scores (Figure 1B). Furthermore, the rotarod test also demonstrated enhanced recovery of hindlimb and tail balance in the Rg1‐EVs group (Figure 1C). Footprint analysis substantiated that mice receiving Rg1‐EVs displayed prominently accelerated recovery of gait and improved motor coordination, relative to their control counterparts (Figure 1D,E). These findings were further corroborated by the outcomes from the bladder reflex recovery and Von Frey filament (VFF) tests (Figure 1F,G). The Louisville Swimming Scale also uncovered that Rg1‐EVs‐treated mice relied less on their forelimbs, demonstrated quicker hindlimb movements, and maintained a diminished body‐water surface angle during swimming (Figure 1H). In addition, the macroscopic examination of the thoracic spinal segment unveiled a smaller lesion area in the Rg1‐EVs group in comparison to the SCI and EVs groups (Figure 1I). Collectively, these behavioral and morphological data confirm that Rg1‐EVs substantially foster the functional recovery of spinal cord‐injured mice.

**Figure 2:**
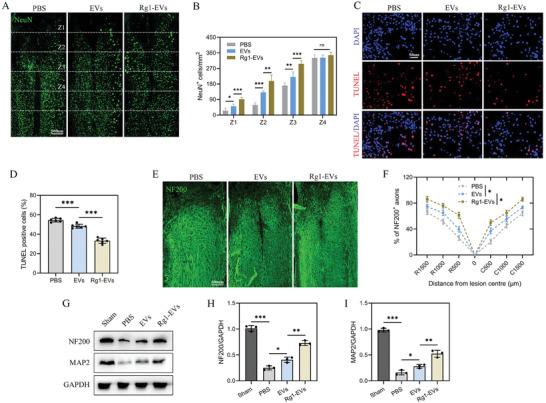
Rg1‐EVs enhance neuroprotective and axonal regenerative effects in SCI mice. A–B) NeuN IF results and statistical analysis (*n* = 6); C–D) TUNEL staining for detection of apoptosis and quantification of TUNEL‐positive cells (*n* = 6); E–F) IF results and statistical analysis of NF200 expression in spinal cord tissue from two groups of mice 28 days following SCI (*n* = 6); G–I) Western blot detection of NF200 and MAP2 in spinal cord tissues on day 28 post‐injury and quantitative analysis (*n* = 3); ^*^ *p <* 0.05; ^**^ *p <* 0.01; ^***^ *p <* 0.001.

The expression of neuronal nuclear antigen (NeuN), serving as a neuronal marker, was assessed through Immunofluorescence (IF) assay in specific regions (Z1–Z4) located at specified distances from lesion boundaries in the spinal cords of experimental mice, as previously described.^[^ ^18^ ^]^ Notably, in regions Z1–Z3, mice receiving Rg1‐EVs demonstrated a notably higher number of NeuN^+^ neuronal cells compared to their counterparts receiving EVs (**Figure** **2**A,B). To validate these observations, TUNEL staining was conducted on day 28 following SCI, which revealed an increased number of TUNEL‐positive neuronal cells in the SCI group. Conversely, both EVs and Rg1‐EVs substantially diminished neuronal apoptosis, with Rg1‐EVs showing the most pronounced neuroprotective effect in minimizing TUNEL‐positive cells (Figure 2C,D). Further anatomical investigations were conducted to evaluate axonal integrity in the SCI lesion core, with NF200‐positive (NF200^+^) axons serving as a marker for axonal regeneration. On day 28 post‐injury, the Rg1‐EVs group displayed a marked elevation in NF200^+^ axons in the core of SCI lesions compared to the EVs group (Figure 2E,F). These morphological data were supported by Western blot results for NF200 and MAP2 (Figure 2G–I). Overall, these results suggest that Rg1‐EVs offer superior neuroprotection, and reduce neuronal apoptosis, and axonal regeneration more effectively than control EVs, thereby facilitating functional recovery in spinal cord‐injured mice.

### 2.3 Rg1‐EVs Modulate Immune Responses and Promote Microglial Polarization

**Figure 3:**
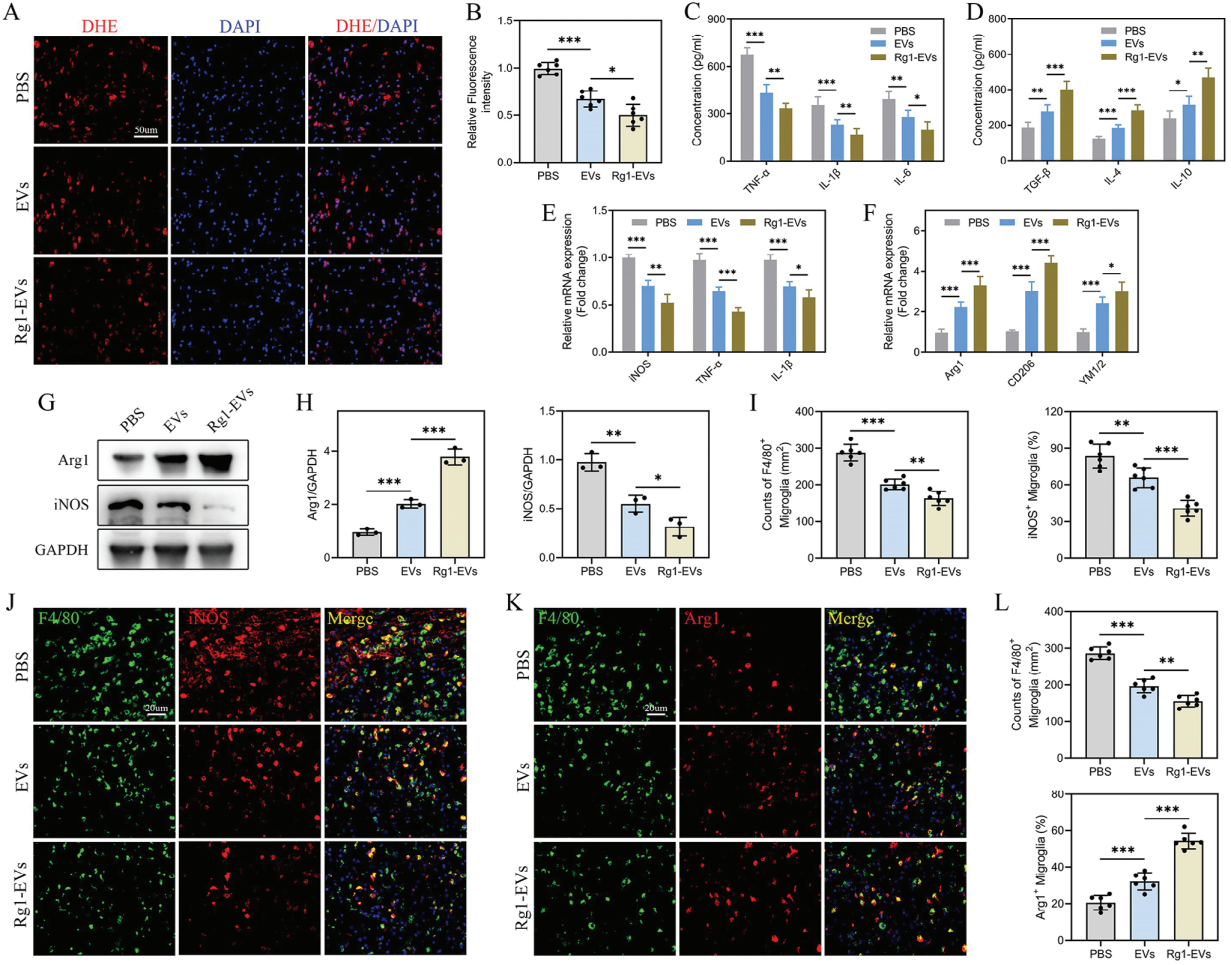
Rg1‐EVs modulate immune microenvironment and microglia polarization in SCI mice. A–B) Evaluation of ROS accumulation in the injured spinal cords of mice from the PBS, EVs, and Rg1‐EVs groups on day 7 post‐injury using DHE staining (*n* = 6); C–D) Statistical analysis of proinflammatory and anti‐inflammatory cytokine concentrations in the injured spinal cords of mice in the three groups on day 7 post‐injury using ELISA (*n* = 3); E,F_ Quantitation of gene expression of M1‐ and M2‐related markers using qRT–PCR (*n* = 3); G–H) Detection of proteins encoded by M1‐ and M2‐related genes using Western blot and quantitative analyses (*n* = 3); I,J) Illustrative immunostaining micrographs of F4/80 (depicted in green) and iNOS (highlighted in red) in the injured area of the spinal cord on day 7 post‐injury, accompanied by an examination of iNOS^+^ microglia in the injured area (*n* = 6); K–L) Illustrative immunostaining micrographs of F4/80 (depicted in green) and Arg1 (highlighted in red) in the injury site of the spinal cord on day 7 post‐injury, accompanied by an examination of Arg1^+^ microglia in the injury site (*n* = 6); ^*^ *p <* 0.05; ^**^ *p <* 0.01; ^***^ *p <* 0.001.

Microglia, pivotal mediators of inflammation in SCI pathology, assume a crucial role in exacerbating secondary injury through inflammatory responses. Prior research has pointed out the capacity of EVs to influence microglial activation and polarization in SCI contexts. Therefore, our investigation explored the effects of Rg1‐EVs on microglial polarization and inflammatory cytokine profiles in the damaged spinal cord. Initially, OS at the injury site was assessed using DHE staining, which indicated a reduction in Reactive Oxygen Species (ROS) accumulation in the injury site after EVs treatment, with Rg1‐EVs demonstrating enhanced effects (**Figure** **3**A,B). ELISA analyses further revealed that treatment with both EVs and Rg1‐EVs groups (versus PBS group) led to noteworthy reductions in proinflammatory cytokines (TNF‐α, IL‐1β, and IL‐6) and increases in anti‐inflammatory cytokines (TGF‐β, IL‐4, and IL‐10), with effects more pronounced in the Rg1‐EVs group (Figure 3C,D). Subsequently, qRT‐PCR analysis confirmed substantial upregulation of M2‐associated genes (Arg1, CD206, YM1/2) and downregulation of M1‐associated genes (iNOS, TNF‐α, IL‐1β) in both EVs and Rg1‐EVs groups (vs PBS group), with Rg1‐EVs leading to a stronger shift toward M2 phenotypes (Figure 3E,F). Western blot findings validated these qRT‐PCR results (Figure 3G,H). Additionally, microglial polarization following SCI was assessed through IF staining for iNOS (M1 phenotype marker), Arg1 (M2 phenotype marker), and F4/80 (microglial marker). Notably, a significant increase in F4/80^+^ cells was observed at the injury site following SCI. However, the inflammatory response was markedly attenuated in both EVs and Rg1‐EVs groups, with the Rg1‐EVs group showing a greater reduction in F4/80^+^ cells compared to those receiving EVs (Figure 3I–L). Furthermore, the levels of Arg1 were elevated, while those of iNOS were attenuated in the Rg1‐EVs group compared with the EVs group (Figure 3I–L). These results suggest that Rg1‐EVs significantly modulate the anti‐inflammatory/proinflammatory phenotypic ratio following SCI and decrease M1‐phenotype microglia and increase M2‐phenotype microglia.

### 2.4 Rg1‐EVs Foster Microglial Polarization In Vitro

The investigation proceeded to clarify the potential replication of in vivo therapeutic effects of Rg1‐EVs on microglial polarization in vitro. Microglial cultures were exposed to lipopolysaccharide (LPS, 1 µg mL^−1^) for 24 h to simulate an SCI‐like inflammatory microenvironment. Following the replacement of the medium, cultures were treated with PBS, EVs, or Rg1‐EVs (100 µg mL^−1^ for 24 h). Both EVs and Rg1‐EVs markedly restricted the secretion of proinflammatory cytokines (TNF‐α, IL‐1β, and IL‐6) and intensified the secretion of anti‐inflammatory cytokines (TGF‐β, IL‐4, and IL‐10), with Rg1‐EVs showing more pronounced effects (Figure S2A,B, Supporting Information). Moreover, qRT‐PCR analysis showed that both treatments downregulated M1‐phenotype markers and upregulated M2‐phenotype markers in microglia, with Rg1‐EVs promoting a more pronounced polarization toward the M2 phenotype (Figure S2C,D, Supporting Information). IF staining further supported these findings, showing a lower percentage of Iba1^+^ iNOS^+^ cells and a higher percentage of Iba1^+^ Arg1^+^ cells in the Rg1‐EVs group relative to the EVs group (Figure S2E–H, Supporting Information). These observations were corroborated by Western blot analysis (Figure S2I–K, Supporting Information). Collectively, these findings indicate that Rg1‐EVs effectively diminish the M1‐phenotype microglia, drive the shift toward M2‐phenotype microglia, and thus curtail inflammation. This confirmation is consistent with the in vivo results.

### 2.5 Rg1‐EVs Regulate Mitochondrial Function and Inhibit OS in Microglia In Vitro

**Figure 4:**
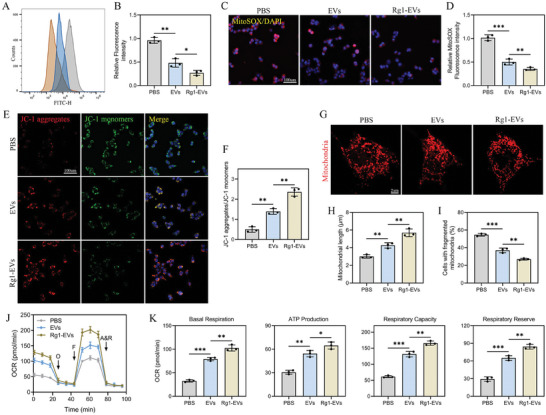
Rg1‐EVs downregulate ROS production and regulate mitochondrial function. A,B) Flow cytometry detection of ROS in microglia treated with PBS, EVs, and Rg1‐EVs and statistical analysis (*n* = 3); C,D) IF staining and quantification of MitoSOX in the three groups (*n* = 3); E,F) Determination of microglial mitochondrial potential by fluorescence microscopy in the three groups using JC‐1 aggregates/JC‐1 monomers and quantification of mitochondrial potential (*n* = 3); G–I) Illustrative images of mitochondrial morphology, quantification of mitochondrial length, and percentage of cells with mitochondrial fragments in the three groups (*n* = 3); J) Detection of OCR in microglia in the three groups using the Seahorse Bioscience XFp analyzer (*n* = 3); K) Determination of mitochondrial activity, including basal respiration, ATP production, respiratory capacity, and respiratory reserve, in the three groups (*n* = 3); ^*^ *p <* 0.05; ^**^ *p <* 0.01; ^***^ *p <* 0.001.

The relationship between microglial polarization and mitochondrial function is well‐documented and was thus a primary focus of this study. We examined the impact of LPS on ROS accumulation, a characteristic feature of the M1 macrophage phenotype. Intriguingly, both EVs and Rg1‐EVs noticeably restricted ROS accumulation in microglia, with Rg1‐EVs exhibiting more pronounced effects (**Figure** **4**A,B). LPS exposure markedly elevated mitochondrial superoxide (MSO) generation, which was also partially counteracted by Rg1‐EVs (Figure 4C,D). Furthermore, JC‐1 staining uncovered a significant recovery in Mitochondrial Membrane Potential (MMP) following treatment with EVs and Rg1‐EVs, with more pronounced improvement observed in the Rg1‐EVs group (Figure 4E,F). Consistent with our previous study,^[^ ^19^ ^]^ LPS treatment shortened mitochondrial length and made them swell, and percentage of cells with fragmented mitochondria was markedly increased as well. Nevertheless, both treatments partially reversed these mitochondrial morphological changes, and the effect of Rg1‐EVs was more pronounced (Figure 4G–I). Furthermore, both treatments significantly increased the Oxygen Consumption Rate (OCR), an indicator of oxidative phosphorylation, with Rg1‐EVs showing more pronounced impacts (Figure 4J). Additionally, both treatments enhanced basal respiration, ATP production, respiratory capacity, and respiratory reserve, with Rg1‐EVs eliciting more potent impacts compared to EVs alone (Figure 4K). These results suggest that Rg1‐EVs diminish ROS production, restore mitochondrial function, and alleviate OS in microglia in vitro.

### 2.6 Rg1‐EVs Promote Functional Recovery and Microglial Polarization Following SCI In Vivo Through the Transfer of MYCBP2

Considering the superior beneficial effects consistently demonstrated by Rg1‐EVs over EVs alone, we hypothesized significant differences in their protein profiles. Proteomic analysis unveiled a marked upregulation of the E3 ubiquitin ligase MYCBP2 in Rg1‐EVs when compared with EVs. To substantiate this observation, we assessed MYCBP2 expression in vitro, noting a significant elevation following Rg1 pretreatment in neuronal cells, isolated EVs, and target microglia (Figure S3A–C, Supporting Information). To elucidate the functional role of MYCBP2 in Rg1‐EVs, we used a lentivirus‐based knockdown approach targeting MYCBP2 in neuronal cells before EV isolation and subsequent coculture with microglia (Figure S3D, Supporting Information). As expected, MYCBP2 expression was significantly reduced in shMYCBP2‐Rg1‐EVs compared with shNC‐Rg1‐EVs (Figure S3E, Supporting Information). Similarly, MYCBP2 protein expression was significantly lower in microglia treated with shMYCBP2‐Rg1‐EVs than in those treated with shNC‐Rg1‐EVs (Figure S3F, Supporting Information). These results suggest that the upregulation and transfer of MYCBP2 by Rg1‐EVs to target microglia are critical mechanisms by which Rg1‐EVs exert their therapeutic effects following SCI.

**Figure 5:**
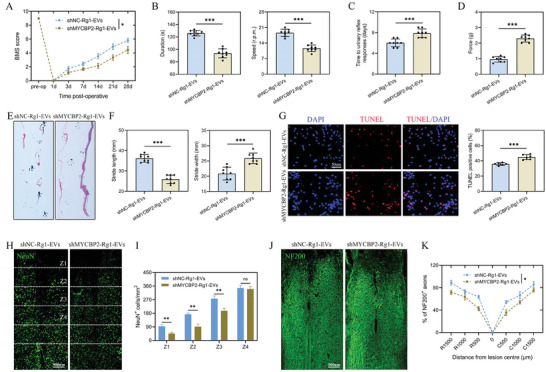
Rg1‐EVs promote functional behavioral recovery and axonal regeneration following SCI via MYCBP2 delivery. A) BMS scores of mice receiving shNC‐Rg1‐EVs and shMYCBP2‐Rg1‐EVs during the recovery period at 28 days post‐injury (*n* = 8); B) Statistical analysis of the results of the rotarod test in the two groups of mice (*n* = 8); C) Analysis of the recovery time of bladder reflex in the two groups of mice (*n* = 8); D) Statistical analysis of the results of the VFF test in the two groups of mice (*n* = 8); E,F) Footprint analysis of the two groups of mice (*n* = 8); G) TUNEL staining to detect apoptosis and quantitative analysis of TUNEL‐positive cells in the two groups (*n* = 6); H,I) IF results and statistical analysis of mature neurons (NeuN) in spinal cord tissues from the two groups of mice 28 days following SCI (*n* = 6); J,K) IF results and statistical analysis of neural axons (NF200) (*n* = 6); ^*^ *p <* 0.05; ^**^ *p <* 0.01; ^***^ *p <* 0.001.

**Figure 6:**
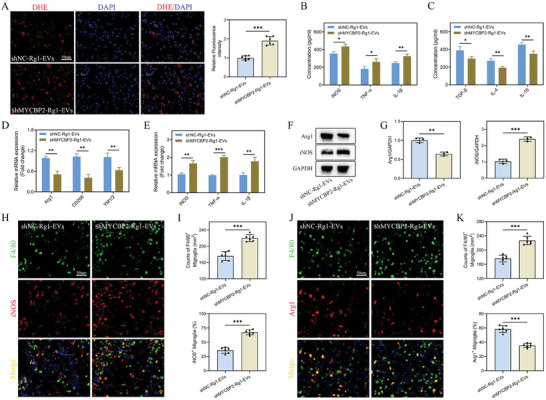
Rg1‐EVs promote microglial M2‐phenotype polarization via MYCBP2 delivery in viv**o**. A. ROS accumulation in the injured spinal cord of mice receiving shNC‐Rg1‐EVs or shMYCBP2‐Rg1‐EVs was detected by DHE staining on day 7 post‐injury (*n* = 6); B,C) ELISA detection of pro‐ and anti‐inflammatory cytokine concentrations in injured spinal cords from the two groups of mice and statistical analysis (*n* = 3); D,E) mRNA expression levels of M1‐ and M2‐related genes detected by qRT–PCR and statistical analyses (*n* = 3); F,G) Expression levels of proteins encoded by M1‐ and M2‐related genes detected by Western blot and quantitative analyses (*n* = 3); H,I) Illustrative immunostaining micrographs of F4/80 (depicted in green) and iNOS (highlighted in red) in the injury site of the spinal cord at 7 days post‐injury, accompanied by an examination of iNOS^+^ microglia in the injury site (*n* = 6); J,K) Illustrative immunostaining micrographs of F4/80 (depicted in green) and Arg1 (highlighted in red) in the injury site of the spinal cord, accompanied by an examination of Arg1^+^ microglia in the injury site on day 7 post‐injury (*n* = 6); ^*^ *p <* 0.05; ^**^ *p <* 0.01; ^***^ *p <* 0.001.

This study further investigated the contribution of MYCBP2 in mediating the effects of Rg1‐EVs on microglial polarization and functional recovery in an SCI model. The spinal cord‐injured mice were treated with shNC‐Rg1‐EVs or shMYCBP2‐Rg1‐EVs. Functional assessments unveiled that mice receiving shMYCBP2‐Rg1‐EVs experienced less favorable recovery outcomes in contrast to those subjected to shNC‐Rg1‐EVs, as reflected by BMS scores, rotarod tests, bladder reflex recovery experiments, VFF tests, and footprint analyses (**Figure** **5**A–F). Additionally, a higher number of TUNEL‐positive cells were observed in mice receiving shMYCBP2‐Rg1‐EVs, suggesting increased neuronal apoptosis (Figure 5G). The shMYCBP2‐Rg1‐EVs treatment attenuated the increase in the number of NeuN^+^ neuronal cells and NF200^+^ axons compared to the shNC‐Rg1‐EVs group (Figure 5H–K). Moreover, the capability of shMYCBP2‐Rg1‐EVs to curb ROS accumulation in the SCI zone was less effective than that of shNC‐Rg1‐EVs (**Figure** **6**A). Importantly, shMYCBP2‐Rg1‐EVs exhibited reduced microglial polarization M2 phenotype, as evidenced by ELISA, qRT‐PCR, and Western blot results (Figure 6B–G). The data from double IF analysis further substantiated these observations (Figure 6H–K). Taken together, these findings suggest that the capability of Rg1‐EVs to encourage functional recovery and microglial M2‐phenotype polarization is associated with MYCBP2.

### 2.7 Rg1‐EVs Promote Microglial Polarization and Orchestrate Mitochondrial Function In Vitro Through the Transfer of MYCBP2

To extend our understanding of the in vivo effects of Rg1‐EVs, we investigated their impact on microglial polarization and mitochondrial function in vitro. Microglia stimulated with LPS were treated with shNC‐Rg1‐EVs or shMYCBP2‐Rg1‐EVs. MYCBP2 knockdown in Rg1‐EVs significantly impaired the ability of Rg1‐EVs to promote the polarization of microglia toward the M2 phenotype (Figure S4A–K, Supporting Information). Moreover, MYCBP2 knockdown in Rg1‐EVs resulted in increased production of ROS and MSO (Figure S5A–D, Supporting Information). The capacity of Rg1‐EVs to enhance MMP and ameliorate changes in mitochondrial morphology was compromised in the presence of MYCBP2 knockdown (Figure S5E–I, Supporting Information). Correspondingly, mitochondrial respiratory function, as assessed by measurements of OCR, basal respiration, ATP production, respiratory capacity, and respiratory reverse, was significantly diminished in microglia subjected to shMYCBP2‐Rg1‐EVs in comparison to those exposed to shNC‐Rg1‐EVs (Figure S5J,K, Supporting Information). These outcomes underscore the critical role of MYCBP2 in mediating the effects of Rg1‐EVs on microglial polarization and mitochondrial function following SCI.

### 2.8 MYCBP2 Binds S100A9 and Regulates its Expression

**Figure 7:**
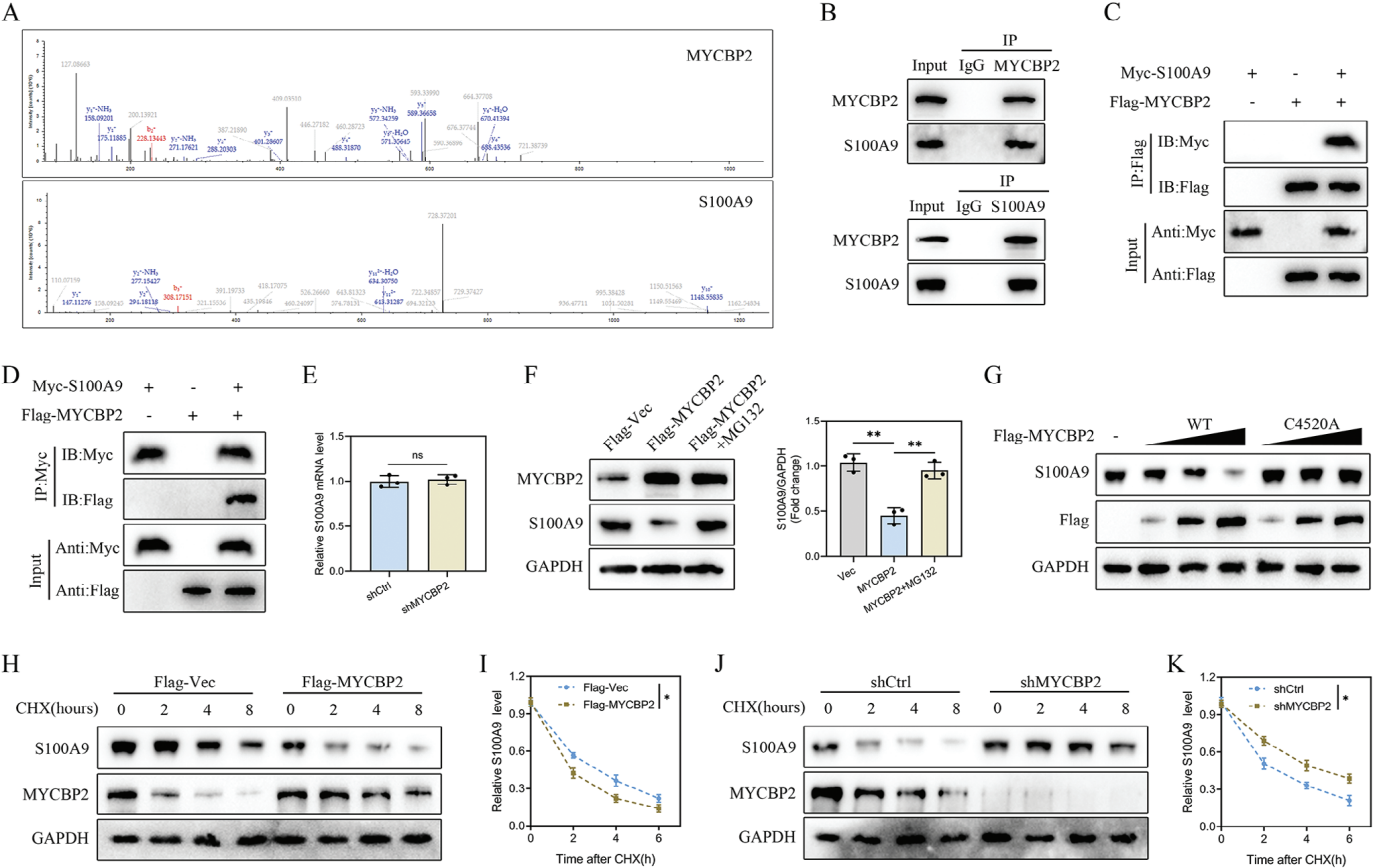
MYCBP2 binds to and regulates S100A9 protein expression. A) Typical peptide sequences of MYCBP2 and S100A9; B. Co‐IP experiments verified the binding between endogenous MYCBP2 and S100A9 in microglia (*n* = 3); C,D) Exogenous overexpression of MYCBP2 and S100A9 in HEK 293T cells similarly demonstrated their exogenous binding (*n* = 3); E) S100A9 mRNA levels in shCtrl‐ and shMYCBP2‐transfected microglia (*n* = 3); F) Analysis of MYCBP2 and S100A9 in Flag‐MYCBP2‐transfected microglia treated with or without the proteasome inhibitor MG132 (*n* = 3); G) Increasing numbers of Flag‐tagged MYCBP2 (WT or C4520A mutant) transfected into HEK 293T cells. Cell lysates were analyzed by immunoblotting with anti‐S100A9 and anti‐Flag (*n* = 3); H,I) S100A9 protein stability was examined after overexpression of MYCBP2 in HEK 293T cells (*n* = 3); J,K) S100A9 protein stability was examined after knockdown of MYCBP2 in HEK 293T cells (*n* = 3); ^*^ *p <* 0.05; ^**^ *p <* 0.01; ^***^ *p <* 0.001.

IP–mass spectrometry (MS) was utilized to identify proteins interacting with MYCBP2, revealing S100A9 as a key interacting protein (**Figure** **7**A). This interaction was further corroborated by Co‐IP experiments, which confirmed that S100A9 specifically precipitated MYCBP2 in microglia, whereas the control immunoglobulin G did not yield such precipitation (Figure 7B). Further verification using reverse Co‐IP illustrated significant precipitation of MYCBP2 by S100A9 in microglia (Figure 7B). Additional Co‐IP assays in HEK 293T cells using epitope‐tagged versions of these proteins corroborated these findings, with Flag‐tagged MYCBP2 efficiently co‐precipitating with Myc‐tagged S100A9 (Figure 7C,D). These data established the interaction between MYCBP2 and S100A9.

Our subsequent investigation delved into the impact of MYCBP2 silencing on S100A9 expression in microglia. MYCBP2 knockdown led to a pronounced increase in S100A9 protein expression without altering its mRNA expression (Figure 7E), suggesting a role for MYCBP2 in regulating S100A9 protein stability. Interestingly, the proteasome inhibitor MG132 counteracted the decline in S100A9 protein levels stimulated by MYCBP2 overexpression (Figure 7F). Moreover, ectopic expression of wild‐type (WT) MYCBP2 restricted S100A9 protein levels, whereas the catalytically inactive C4520A mutant of MYCBP2 did not produce the same effect (Figure 7G). In experiments using the protein synthesis inhibitor cycloheximide, MYCBP2 overexpression prominently accelerated S100A9 degradation, while MYCBP2 knockdown markedly hindered S100A9 degradation (Figure 7H–K). These results establish that MYCBP2 directly interacts with and influences the stability and degradation of S100A9, highlighting a regulatory mechanism in microglia.

### 2.9 MYCBP2 Promotes S100A9 Ubiquitination and Degradation

**Figure 8:**
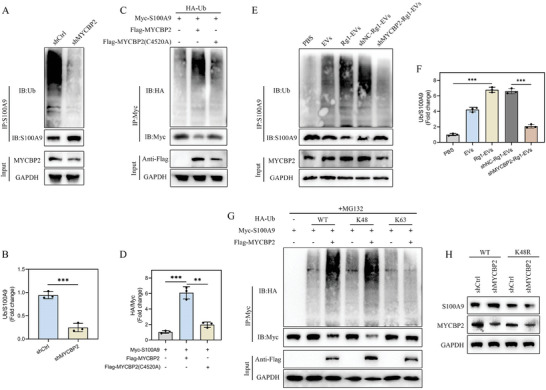
MYCBP2 promotes S100A9 ubiquitination. A) Immunoprecipitation of lysates from microglial cells transfected with shCtrl or shMYCBP2 and treated with MG132 prior to being harvested and evaluated with the indicated antibodies; B) Quantification of the relative levels of ubiquitin‐S100A9 (*n* = 3); C) Detection of endogenous ubiquitination levels of S100A9 protein after overexpression of MYCBP2 and its enzymatically active point mutant MYCBP2 C4520A in microglia; D) Quantification of the relative levels of ubiquitin‐S100A9 (*n* = 3); E) Immunoprecipitation of spinal cord lysates from the indicated groups of mice after injury with anti‐S100A9, followed by immunoblotting with anti‐Ub and anti‐S100A9; F) Quantification of the relative levels of ubiquitin‐S100A9 (*n* = 3); G) Quantification of HEK 293T cells co‐transfected with Flag‐MYCBP2, Myc‐S100A9, and the indicated HA‐Ub, K48‐only, or K63‐only plasmids, and assessment of S100A9 ubiquitination (*n* = 3); H) HEK 293T cells transfected with ubiquitin WT or ubiquitin K48R were cultured in the presence of shCtrl or shMYCBP2 for 72 h. Cell lysates were assessed by immunoblotting with anti‐S100A9 and anti‐MYCBP2 (*n* = 3); ^**^ *p <* 0.01; ^***^ *p <* 0.001.

Ubiquitination, a crucial post‐translational modification for proteasomal degradation, was investigated in the context of MYCBP2, an E3 ubiquitin ligase, and its regulatory effects on S100A9 stability. The observations indicated that MYCBP2 knockdown reduced S100A9 ubiquitination and simultaneously increased S100A9 protein levels (**Figure** **8**A,B). To substantiate the impact of MYCBP2 on S100A9 ubiquitination, co‐transfection experiments were performed in HEK 293T cells using Myc‐S100A9, Flag‐tagged MYCBP2 (WT or C4520A mutant), and hemagglutinin‐tagged ubiquitin (HA‐Ub). The results demonstrated that WT MYCBP2 overexpression enhanced S100A9 ubiquitination, an effect not observed with the C4520A mutant (Figure 8C,D). In the context of SCI, the ubiquitination and subsequent degradation of S100A9 were noticeably boosted in the spinal cord tissues of mice receiving Rg1‐EVs compared with those receiving EVs alone. Conversely, mice treated with shMYCBP2‐Rg1‐EVs displayed reduced S100A9 ubiquitination and degradation in the spinal cord tissues compared with their counterparts receiving shNC‐Rg1‐EVs (Figure 8E,F).

The research focus shifted toward assessing the influence of MYCBP2 on the polyubiquitination of S100A9. The results elucidated that MYCBP2 selectively cleaved the K48‐linked polyubiquitin chain but not the K63‐linked polyubiquitin chain (Figure 8G). The introduction of a K48‐resistant form of ubiquitin in MYCBP2‐knockdown HEK 293T cells substantiated the dependency of S100A9 degradation on K48‐linked polyubiquitination. This intervention effectively counteracted the rise in S100A9 levels stimulated by MYCBP2 knockdown (Figure 8H). In summary, these data indicate that MYCBP2 promotes the ubiquitination and degradation of S100A9 in microglia.

### 2.10 MYCBP2‐Harboring Rg1‐EVs Promote Microglial Polarization and Regulate Mitochondrial Function by Enhancing S100A9 Degradation

**Figure 9:**
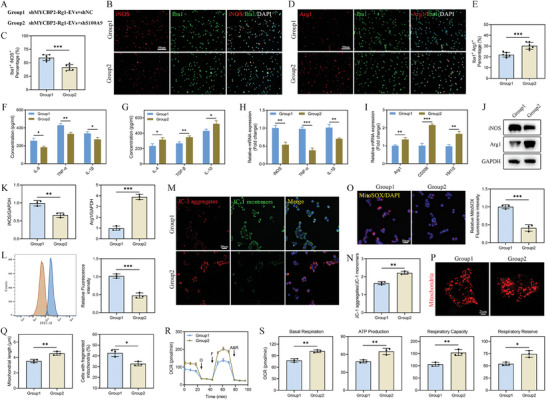
EVs containing MYCBP2 regulate M2‐phenotype microglial polarization and mitochondrial function through S100A9 degradation. A series of in vitro rescue experiments were performed to validate the functional role of MYCBP2‐containing EVs in microglia. Rescue experiments of MYCBP2‐containing depleted EVs were carried out by ectopic knockdown of S100A9. A) Detailed information of the experimental groups in this part of the experiment; B,C) IF staining of Iba1 and iNOS in microglial cells of both groups and statistical analysis (*n* = 6); D,E) IF staining of Iba1 and Arg1 in microglial cells of both groups and statistical analysis (*n* = 6); F,G) ELISA of pro‐ and anti‐inflammatory cytokines in both groups (*n* = 3); H,I) Detection of mRNA expression levels of M1‐ and M2‐related genes by qRT–PCR (*n* = 3); J,K) Detection of proteins encoded by M1‐ and M2‐associated genes by Western blot and statistical analyses (*n* = 3); L) Detection of ROS accumulation in microglial cells of the two groups by flow cytometry and statistical analyses (*n* = 3); M,N) Determination and quantification of mitochondrial potential by JC‐1 aggregates/JC‐1 monomers (*n* = 3); O) IF staining and quantification of MitoSOX in both groups (*n* = 3); P,Q) Illustrative images of mitochondrial morphology, quantification of mitochondrial length, and the percentage of cells with mitochondrial debris in both groups (*n* = 3); R) Detection of OCR in microglial cells in both groups using a Seahorse Bioscience XFp analyzer (*n* = 3); S) Determination of mitochondrial activity, including basal respiration, ATP production, respiratory capacity, and respiratory reserve, in both groups (*n* = 3); ^*^ *p <* 0.05; ^**^ *p <* 0.01; ^***^ *p <* 0.001.

The study further clarified the involvement of the MYCBP2–S100A9 interaction in orchestrating microglial polarization and mitochondrial function. In in vitro rescue experiments, we silenced S100A9 in microglia and observed that S100A9 knockdown significantly reversed the effects of shMYCBP2‐Rg1‐EVs on microglial polarization and augmented polarization toward the M2 phenotype (**Figure** **9**A–K). Additionally, the targeted reduction of S100A9 expression attenuated the elevation in ROS accumulation and the decline in MMP, both of which were initially exacerbated by shMYCBP2‐Rg1‐EVs (Figure 9L–N). Furthermore, S100A9 knockdown fostered the capability of shMYCBP2‐Rg1‐EVs to reduce MSO generation and reverse changes in mitochondrial morphology (Figure 9O–Q). In addition, S100A9 silencing abolished the reductions in OCR, basal respiration, ATP production, respiratory capacity, and respiratory reverse previously induced by shMYCBP2‐Rg1‐EVs (Figure 9R–S). These findings indicate that MYCBP2‐containing EVs regulate microglial polarization and mitochondrial function through the degradation of S100A9.

### 2.11 Rg1‐EVs Promote Motor Function Recovery and Microglial Polarization In Vivo Through Ubiquitin‐Mediated Degradation of S100A9

**Figure 10:**
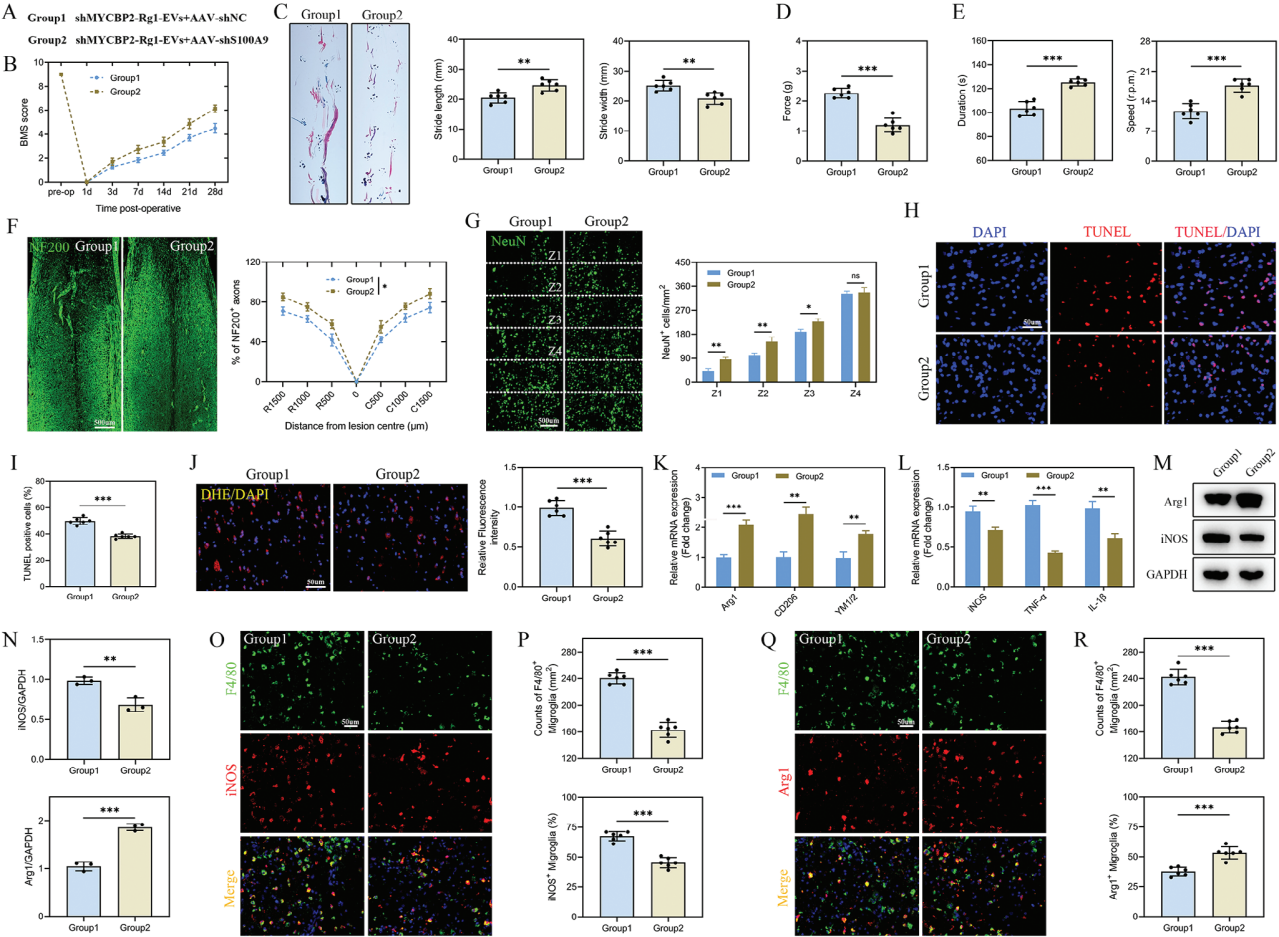
EVs containing MYCBP2 promote functional recovery, axonal regeneration, and microglial M2‐phenotype polarization following SCI by degrading S100A9. A) Detailed information of experimental groups in this part of the experiment; B) BMS scores of the two groups of mice during the recovery period at 28 days post‐injury (*n* = 6); C) Footprint analyses of the two groups (*n* = 6); D) Statistical analysis of the results of the VFF test in the two groups of mice (*n* = 6); E) Statistical analysis of the results of rotarod tests in the two groups of mice (*n* = 6); F) IF results and statistical analysis of neuronal axons (NF200) in spinal cord tissues from the two groups of mice 28 days following SCI (*n* = 6); G) IF results and statistical analysis of mature neurons (NeuN) in spinal cord tissues from the two groups of mice 28 days following SCI (*n* = 6); H,I) Detection of apoptosis in the two groups of cells by TUNEL staining and quantitative analysis of TUNEL‐positive cells (*n* = 6); J) DHE staining to detect ROS accumulation in the injured spinal cord from the two groups of mice (*n* = 6); K,L) Detection of mRNA expression levels of M1‐ and M2‐related genes by qRT–PCR and statistical analysis (*n* = 3); M,N) Detection of proteins encoded by M1‐ and M2‐related genes by Western blot and quantitative analysis (*n* = 3); O,P). IF results and statistical analysis of the SCI area on day 7 post‐injury for F4/80 (depicted in green) and iNOS (highlighted in red), accompanied by an examination of iNOS^+^ microglia in the injury site (*n* = 6); Q,R) Illustrative immunostaining micrographs of F4/80 (depicted in green) and Arg1 (highlighted in red) in the SCI zone on day 7 post‐injury, accompanied by an examination of Arg1^+^ microglia in the injury site (*n* = 6); ^**^ *p <* 0.01; ^***^ *p <* 0.001.

The role of S100A9 in the neuroprotective effects of Rg1‐EVs was further illustrated in an experimental model, where spinal cord‐injured mice were injected with AAVs to specifically knock down S100A9 in microglia and then administered shMYCBP2‐Rg1‐EVs. Motor function assessments, including BMS scores, footprint analyses, VFF test, and rotarod tests, revealed that functional recovery was significantly enhanced in mice receiving shMYCBP2‐Rg1‐EVs + shS100A9 compared with those treated with shMYCBP2‐Rg1‐EVs + shNC (**Figure** **10**A–E). The knockdown of S100A9 notably negated the decrease in the number of NeuN^+^ neuronal cells and NF200^+^ axons typically induced by shMYCBP2‐Rg1‐EVs (Figure 10F,G). Further, treatment with shMYCBP2‐Rg1‐EVs + shS100A9 resulted in a more pronounced decrease in TUNEL‐positive cells than treatment with shMYCBP2‐Rg1‐EVs + shNC (Figure 10H,I). Finally, treatment with shMYCBP2‐Rg1‐EVs + shS100A9 significantly halted ROS production and augmented microglial polarization toward the M2 phenotype than the control treatment (Figure 10J–R). These data suggest that the neuroprotective and functional recovery‐promoting effects of Rg1‐EVs in vivo are mediated through the ubiquitin‐dependent degradation of S100A9, facilitating the transition of microglia to the anti‐inflammatory M2 phenotype.

## 3 Discussion

SCI leads to severe neurological impairments and remains a significant therapeutic challenge. The polarization state of microglia and OS are important factors in the pathophysiology of SCI, which influence the microenvironment and subsequent nerve regeneration.^[^ ^20^ ^]^ Therefore, modulating microglial polarization and OS is crucial for fostering axonal regeneration and motor recovery following SCI. The present study reveals the potential mechanism by which Rg1‐EVs promote functional recovery in the injured spinal cord. Our findings demonstrated that Rg1‐EVs significantly supported the recovery of motor functions in mice by enhancing microglia's polarization toward the M2 phenotype and reducing OS. These effects are primarily driven by the delivery of MYCBP2 into microglia, which promotes the ubiquitination and degradation of S100A9. This degradation process crucially facilitated the M2 polarization state and hindered OS accumulation. The findings of this study highlight the therapeutic potential of Rg1‐EVs as mediators of neuroprotection and neural repair, providing valuable insights into their role in ameliorating the adverse effects of SCI.

Recent advancements have underscored the importance of EVs in biomedical research, particularly for their pivotal roles in intercellular communication and their therapeutic potential in tissue regeneration and repair. As nanoscale vesicles derived from cells, EVs encapsulate and transport a range of biologically active substances, including proteins, RNAs, small molecules, and other biomolecules, to recipient cells. This intercellular EV pathway facilitates the modulation of the function and behavior of recipient cells.^[^ ^21^ ^]^ In the context of SCI therapy, EVs have demonstrated capabilities to promote neuronal survival, bolster nerve regeneration, and reduce inflammatory responses.^[^ ^22^ ^]^ The therapeutic efficacy of EVs can be further augmented through various pretreatment strategies, such as genetic engineering, pharmacological treatment, or biological modifications.^[^ ^23^, ^24^ ^]^ For example, the incorporation of specific growth factors, anti‐inflammatory factors, or other neuroprotective proteins can prime EVs for enhanced neuroprotection and repair.^[^ ^25^, ^26^ ^]^ Notably, EVs derived from hypoxically cultured mesenchymal stem cells (MSCs) have been shown to modulate microglial polarization from M1 to M2 phenotypes, thereby aiding in the repair of traumatic SCI.^[^ ^27^ ^]^ Additionally, EVs from hypoxic human umbilical vein endothelial cells have been reported to enhance the angiogenic capabilities of MSCs, further contributing to spinal cord repair.^[^ ^28^ ^]^ Furthermore, pretreatment of MSC‐derived EVs with melatonin facilitates the repair of traumatic SCI by stabilizing nuclear factor erythroid 2‐related factor 2 (Nrf2).^[^ ^29^ ^]^

Ginsenoside Rg1 is recognized for its broad spectrum of pharmacological properties, including anti‐inflammatory, antioxidant, antiapoptotic, anti‐excitotoxic, and neuroprotective activities, which contribute to its functional effects across various pathological conditions. For example, Rg1 reduces OS and downregulates the Akt/mTOR signaling pathway, thus attenuating cognitive dysfunction induced by D‐galactose and preventing senescence in neural stem cells in mice.^[^ ^30^ ^]^ Additionally, Rg1 ameliorates cerebral ischemia–reperfusion injury by targeting Toll‐like receptor 4 in microglia,^[^ ^31^ ^]^ and it supports functional recovery following SCI by inhibiting OS and inflammation via the Nrf2/HO‐1 signaling pathway.^[^ ^16^ ^]^ Despite these insights, the application of Rg1 in preconditioning EVs remains underexplored, particularly in the context of CNS injuries. Rg1, as an active ingredient, not only promotes the secretion of EVs from neuronal cells, but also improves the therapeutic effects of EVs secreted by pretreated neuronal cells, rendering them more effective in regulating cell function and protecting cells from damage. In addition, Rg1 pretreatment may enhance the stability and targeting specificity of EVs, suggesting that EVs from pretreated sources exhibit more controlled and specific distribution and function in vivo, thus contributing to improved efficacy and reduced adverse effects. Oral Rg1 undergoes enzymatic and first‐pass effects in the gut, resulting in low actual absorption. Injectable Rg1‐EVs reach the target tissues faster and provide more rapid pharmacological effects. This study's novel findings demonstrate that Rg1‐EVs surpass EVs alone in promoting neurological recovery following SCI in mice.

Microglia, the primary immune cells in the CNS, are known to rapidly activate and undergo polarization after injury. In the context of SCI, microglia exhibit dual phenotypic states: proinflammatory M1 and anti‐inflammatory M2 phenotypes, which play critical roles in influencing the SCI microenvironment, respectively.^[^ ^32^ ^]^ Initially, microglia tend to adopt a proinflammatory M1 phenotype, releasing large amounts of proinflammatory cytokines that can exacerbate injury and hinder neuronal repair and regeneration. However, over the course of recovery, a transition toward an anti‐inflammatory and reparative M2 phenotype often occurs, which aids in debris removal from the injury site and nerve regeneration. Strategies to promote M2 phenotype polarization of microglia include pharmacological treatments, gene therapy, MSC transplantation, and EV administration.^[^ ^33^, ^34^, ^35^ ^]^ Consistent with existing studies, our study confirms that treatment with EVs and Rg1‐EVs facilitates functional recovery and polarizes microglia to the M2 phenotype, underscoring the therapeutic potential of EVs in managing and potentially ameliorating the effects of SCI.

OS is characterized by the excessive accumulation of free radicals and other oxidative substances that can damage cellular components, such as cell membranes, proteins, and DNA, thereby triggering cell death and tissue damage.^[^ ^36^ ^]^ This process is particularly exacerbated in SCI, where elevated levels of OS contribute to ongoing cellular dysfunction. The accumulation of free radicals and other oxidizing substances not only triggers enhanced inflammatory responses by activating and releasing inflammatory mediators, but also causes direct damage to newly formed neuronal cells and axons, thereby impeding their growth and integration at the injury site. In addition, OS can disrupt critical signaling pathways, including those related to nerve growth factors essential for nerve regeneration, and impede the repair processes necessary for neural recovery. Research indicates that sirtuin 1 can attenuate OS effects by deacetylating p66Shc, thus preserving endothelial barrier function.^[^ ^37^ ^]^ Another study involving a metal–iridium complex targeting antioxidant 1 protein demonstrated significant alleviation of SCI by suppressing OS‐related ROS.^[^ ^38^ ^]^ In this study, treatments with EVs and Rg1‐EVs were found to diminish ROS production and OS at the injury site, highlighting their potential therapeutic benefits in managing SCI‐induced oxidative damage.

Interestingly, Rg1‐EVs demonstrated a superior capacity to induce microglia polarization toward the anti‐inflammatory M2 phenotype and mitigate the deleterious effects of OS compared to EVs alone. This enhanced potency of Rg1‐EVs can be attributed to their specific protein cargo, which enables the targeted delivery of biologically active molecules to recipient cells. For instance, activated astrocytes have been suggested to stimulate microglial activation and neuronal apoptosis following SCI through EV‐mediated transportation of C‐C motif chemokine ligand 2,^[^ ^39^ ^]^ whereas EV‐mediated delivery of TGF‐β1 augments M2 macrophage polarization in mice.^[^ ^40^ ^]^ Similarly, Wei et al. reported that EV‐mediated delivery of macrophage migration inhibitory factors contributed to temozolomide resistance in gliomas by downregulating the tissue inhibitor of metalloproteinase 3 and activating the PI3K/Akt signaling pathway.^[^ ^41^ ^]^ In light of these findings, we focused on the role of specific protein cargos in Rg1‐EVs. Our proteomic analyses revealed a significant enrichment of MYCBP2 in these EVs, which corresponded to their potent anti‐inflammatory responses and capability to counteract OS in microglia. The critical role of MYCBP2 was further underscored by experiments showing that its depletion from Rg1‐EVs partially diminished their therapeutic effects, emphasizing the importance of specific protein cargos in optimizing the functional impact of EVs in SCI recovery.

MYCBP2 is a Myc‐bound protein with multiple structural domains and functional regions that serve as cell signaling hubs. This complex molecular feature enables MYCBP2 to regulate a wide range of cellular processes, including neuronal development, cytoskeletal dynamics, and axonal degeneration.^[^ ^42^, ^43^ ^]^ Importantly, MYCBP2 exhibits atypical RING ubiquitin ligase activity, which plays a critical role in inhibiting the p38/MAPK and JNK pathways, thereby influencing the cytoskeletal dynamics of axonal development and synaptic growth.^[^ ^44^, ^45^ ^]^ Moreover, its role extends to promoting chemoresistance and suppressing neuronal autophagy via the targeted ubiquitination of F‐box and WD repeat‐containing protein 7 and Unc‐51‐like kinase 1.^[^ ^46^, ^47^ ^]^ As an E3 ubiquitin ligase, MYCBP2 has been implicated in cell proliferation, signaling, and other pivotal biological processes. In the present study, the overexpression of MYCBP2 in Rg1‐EVs has been identified as a significant factor influencing microglial polarization. Although prior research has linked mutations or dysregulation of MYCBP2 with various disorders, such as neurodegenerative diseases and cancers, its specific functions in the CNS, especially in the modulation of microglial polarization following SCI, have not been fully elucidated.

In our study, we utilized IP–MS to explore the protein–protein interactions and potential mechanisms of the regulatory role of MYCBP2 in the polarization and OS dynamics of M2‐phenotype microglia. Notably, our findings highlighted the potential involvement of S100A9, a member of the S100 family of calcium‐binding proteins known for its role in immune regulation, inflammation, and microglial activation, as a possible interacting partner of MYCBP2 in microglia. S100A9 is involved in CNS homeostasis and the cellular response to injury. For instance, the use of specific S100A9 blockers has been shown to reduce neutrophils and monocyte/macrophage presence in the myocardium, thereby fostering cardiac function recovery after myocardial infarction.^[^ ^48^ ^]^ Similarly, inhibition of S100A9 enhances neuroprotection and functional recovery following traumatic brain injury.^[^ ^49^ ^]^ and has been associated with suppressed neutrophil infiltration and alleviated inflammatory responses, ultimately supporting neuronal viability and motor function recovery following SCI.^[^ ^50^ ^]^ This study provides evidence of the direct interaction between MYCBP2 and S100A9, which facilitates the ubiquitin‐mediated degradation of S100A9 in microglia and the injured spinal cord. Notably, the depletion of S100A9 in mice also augmented M2‐phenotype microglial polarization in microglia and resulted in a significant reduction in OS. These findings underscore the significance of S100A9 degradation in the beneficial effects of Rg1‐EVs on microglial polarization. These results suggest that MYCBP2 might regulate microglial polarization by modulating S100A9 stability. While this research highlighted the potential of Rg1‐EVs as a promising strategy for SCI treatment, the development of EVs as multifunctional delivery systems for MYCBP2 or S100A9 shRNA emerges as a promising therapeutic strategy. Indeed, our previous research has demonstrated that the delivery of RNAs, proteins, and drug molecules via EVs, presents a viable alternative to conventional drug delivery methods, especially in SCI treatments.^[^ ^51^ ^]^ Future studies should consider the design of EVs tailored for delivering MYCBP2 or S100A9 shRNA to further enhance therapeutic outcomes.

In conclusion, this study provides new insights into the critical role of Rg1‐EVs in SCI treatment. These findings shed light on novel therapeutic targets in the pathological processes associated with SCI, particularly the regulation of M2‐phenotype microglial polarization and OS reduction through MYCBP2‐mediated mechanisms. These pathways are crucial for neuroprotection and the enhancement of functional recovery following SCI. However, the practical use of EVs in clinical settings is currently limited by their low production yield and inconsistent quality. The insights gained from this study suggest promising avenues for enhancing EV‐based therapies for SCI.

## 4 Experimental Section

### Cell Culture

Primary neuronal cells were isolated under aseptic conditions from the spinal cords of neonatal C57BL/6 mice (1–3 days old). The spinal columns were rapidly excised and immediately submerged in chilled saline within a petri dish. Under stereomicroscopic guidance, spinal cord tissues were carefully separated from the vertebral columns using a fine needle. These tissues were then sectioned into ≈1 mm^3^ blocks and subjected to enzymatic dissociation for 20 min in a culture medium containing 0.25% trypsin (Thermo Fisher Scientific, USA) and 0.1% DNase I (Sigma–Aldrich) under continuous agitation. The neuronal cells were then isolated and enriched through successive rounds of centrifugation and resuspension. After centrifugation, the cells were cultured on plates pre‐coated with polylysine and maintained in a Neurobasal medium supplemented with 2% B‐27 (Thermo Fisher Scientific), 2 mM L‐glutamine (Gibco), and 1% penicillin–streptomycin solution. Half of the culture medium was refreshed every other day to maintain optimal growth conditions. The purity and viability of the neuronal cultures were regularly monitored using immunocytochemical staining.

Primary microglia were isolated using neonatal mice as per established protocols. Brain tissues from these mice were dissociated. Subsequently, 5 × 10^7^ cells were seeded into a 75 cm^2^ flask coated with poly‐L‐lysine (Corning). These cells were cultured in Dulbecco's modified Eagle's medium supplemented with 10% fetal bovine serum (Gibco) and penicillin–streptomycin. The cultures were maintained at 37 °C in a humidified incubator with 5% CO_2_ and 95% air. On day 10, the microglia were agitated at 250 rpm for 90 min at 37 °C to facilitate detachment. Subsequently, the cells were transferred to a 24‐well plate for further experimental use.

### Isolation and Characterization of EVs

Primary neuronal cells were cultured with or without 40 µM ginsenoside Rg1. After a 48 h incubation, the conditioned medium was collected and initially centrifuged at 300 ×g and then at 2000 × g for 10 min at each step to precipitate cells and large debris. The resulting supernatants were then filtered through a 0.22 µm filter to eliminate residual cell debris. The filtrated supernatants were further centrifuged at 140 000 g for 70 min at 4 °C. The resulting pellets were then resuspended in phosphate‐buffered saline (PBS) and subjected to another round of centrifugation under the same conditions. The purified EVs were then resuspended in PBS and stored at −80 °C for further analysis. The morphological characteristics of the EVs were examined using TEM.^[^ ^51^ ^]^ The size distribution was evaluated through NTA.^[^ ^51^ ^]^ EV‐specific surface markers, including CD81, CD63, CD9, ALIX, and TSG101, were verified using Western blot assay.

### Plasmid Design and Transfection Procedures

Flag‐tagged constructs of MYCBP2, its C4520A mutant variant, and Myc‐tagged S100A9 were synthesized by inserting their respective N‐terminal Flag or Myc epitope sequences supplied by Genebay Biotech. Furthermore, plasmids harboring hemagglutinin (HA)‐tagged ubiquitin variants K63 and K48, along with lentiviral vectors encoding short hairpin RNAs (shRNAs) targeting MYCBP2, were also procured from Genebay Biotech. A lentiviral construct without a specific target sequence served as the negative control (NC). These constructs were transfected using Lipofectamine 3000 (Invitrogen) following the manufacturer's protocol. For in vivo experiments, adeno‐associated virus (AAV) vectors (F4/80) expressing a shRNA targeting S100A9 or a shRNA NC were developed by Hanbio Biotechnology. Following SCI induction, mice were positioned on a stereotactic device, and a total of 2 × 10^9^ viral genomes of the prepared vectors were injected into the spinal cord at an infusion rate of 0.25 µL min^−1^.

### RNA Isolation and Quantitative Real‐Time PCR (qRT‐PCR)

Total RNA was extracted using TRIzol reagent (Invitrogen) following the manufacturer's protocol. Reverse transcription of the extracted RNA was then implemented using a reverse transcription system (Toyobo). The synthesized cDNA was quantified using the ABI 7900 Fast Real‐Time PCR System (Applied Biosystems) equipped with SYBR Green PCR Master Mix (Applied Biosystems) to ensure accurate measurement of gene expression. The expression of target genes was normalized against internal controls, and the relative expression levels were calculated employing the 2^−ΔΔCT^ method for quantitative analysis. Details regarding the specific primer sequences used are available upon request.

### Measurement of OCR

The OCR was quantified utilizing the Seahorse XF96 Metabolic Flux Analyzer (Seahorse Biosciences). The measurement protocol involved the sequential addition of metabolic inhibitors and uncouplers to assess mitochondrial function. Initially, oligomycin, an ATP synthase inhibitor, was added at a concentration of 2 µmol L^−1^ to inhibit mitochondrial ATP production. This process was followed by the addition of carbonyl cyanide 4‐(trifluoromethoxy) phenylhydrazone, a mitochondrial uncoupler, at 1 µmol L^−1^ to assess maximal respiratory capacity. Finally, rotenone and antimycin A, inhibitors of mitochondrial complexes I and III, respectively, were each added at 1 µmol L^−1^ to evaluate non‐mitochondrial respiration. Data were quantified and analyzed using the XFe Wave software (Seahorse Biosciences), which facilitated the calculation of key respiratory parameters, including basal respiration, ATP‐linked respiration, maximal respiratory capacity (maximum electron transport chain activity), and spare respiratory capacity (adaptability of the system to heightened energy demand) following the guidelines provided by the manufacturer.

### Evaluation of MMP and ROS

MMP was assessed using the JC‐1 Assay Kit (Beyotime, China). Additionally, mitochondrial ROS were determined using the MitoSOX Red mitochondrial superoxide  indicator (Molecular Probes, USA). Confocal microscopy was utilized to visualize the oxidative state of the mitochondria. ROS accumulation was quantified with a ROS Detection Kit (Beyotime) using 2′,7′‐dichlorofluorescein diacetate as a detection probe, followed by flow cytometric analysis.

### Examination of Mitochondrial Structure

Microglia were incubated for 30 min at 37 °C with 100 nM MitoTracker Red CMXRos (Invitrogen) in accordance with the manufacturer's instructions. Following this, mitochondrial structures were visualized using a confocal microscope. Quantitative analysis of these images was performed using ImageJ software to evaluate changes in mitochondrial morphology and integrity.

### Western Blot

Cells and spinal cord tissues were lysed using radioimmunoprecipitation assay (RIPA) lysis buffer (Beyotime). The proteins in the lysates were resolved by sodium dodecyl sulfate–polyacrylamide gel electrophoresis and electroblotted onto polyvinylidene fluoride membranes. The membranes were blocked for 2 h at ambient temperature in 5% skim milk solution diluted in 0.1% Tween‐20‐containing Tris‐buffered saline. The blocked membranes were incubated overnight at 4 °C with specific primary antibodies. After washing with Tris‐buffered saline containing 0.1% Tween‐20, the membranes were incubated for 2 h with horseradish peroxidase‐conjugated secondary antibodies (Jackson ImmunoResearch) at a dilution of 1:10000. Protein bands were visualized using the PowerOpti‐ECL detection system (Thermo Fisher Scientific). The following primary antibodies were used: anti‐CD9 (Santa Cruz Biotechnology, 1:500), anti‐CD63 (Abcam, 1:2000), anti‐CD81 (Santa Cruz Biotechnology, 1:500), anti‐ALIX (Santa Cruz Biotechnology, 1:1000), anti‐TSG101 (Abcam, 1:2000), anti‐Calnexin (Cell Signaling Technology, 1:1000), anti‐MAP2 (Proteintech, 1:10 000), anti‐NF200 (Abcam, 1:5000), anti‐iNOS (Abcam, 1:1000), anti‐Arg1 (Cell Signaling Technology, 1:1000), anti‐MYCBP2 (Abcam, 1:5000), anti‐S100A9 (Abcam, 1:1000), anti‐GAPDH (Proteintech, 1:20 000), anti‐Flag‐Tag (Cell Signaling Technology, 1:2000), anti‐HA‐Tag (Cell Signaling Technology, 1:2000), and anti‐Myc‐Tag (Cell Signaling Technology, 1:2000).

### Immunoprecipitation (IP)

Microglia and HEK 293T cells were lysed using RIPA buffer containing a cocktail of protease inhibitors. Protein concentrations in these lysates were quantified using the BCA Protein Assay Kit (Thermo Fisher). Lysates were incubated for 1 h with protein A/G‐agarose beads (Santa Cruz Biotechnology), followed by a 2 h incubation with protein A/G‐agarose beads to capture the antibody‐antigen complexes. The beads were then rinsed five times using RIPA buffer to remove unbound substances. The immune complexes were eluted by boiling and subsequently analyzed by Western blot.

### Co‐IP Assay

The Co‐IP assay was carried out utilizing the Pierce Co‐IP Kit (Thermo Fisher Scientific) as per the provided protocol. Lysates from microglia or HEK 293T cells were mixed with antibodies pre‐bound to agarose beads and incubated overnight at 4 °C with gentle rotation. After incubation, the beads were washed multiple times with lysis buffer to remove non‐specifically bound proteins. The proteins bound to the beads were then eluted with elution buffer and prepared for further analysis via Western blot assay.

### Immunofluorescence (IF) Assay

Cells or tissue sections were fixed using 4% paraformaldehyde and subsequently permeabilized using 0.3% Triton X‐100. The samples were then blocked with 5% bovine serum albumin to prevent non‐specific binding. Primary antibodies were applied and incubated overnight at 4 °C. Following primary antibody incubation, samples were treated with fluorophore‐conjugated secondary antibodies (Jackson ImmunoResearch, diluted at 1:200) and counterstained with 4′,6‐diamidino‐2‐phenylindole for nuclear visualization. Fluorescent images were acquired using a fluorescence microscope to evaluate the localization and expression levels of the target proteins. The following primary antibodies were used: anti‐MAP2 (Novus, 1:200), anti‐NF200 (Abcam, 1:200), anti‐NeuN (Abcam, 1:400), anti‐F4/80 (Abcam, 1:200), anti‐iNOS (Abcam, 1:500), anti‐Arg1 (Cell Signaling Technology, 1:500), and anti‐Iba1 (Thermo Fisher Scientific, 1:500).

### Development of the SCI Model

The experimental procedures utilized in this study were approved by the Animal Research Committee of the First Affiliated Hospital of the University of Science and Technology, China (Granted Number:2024‐N(A)−17). A mouse model of SCI was established using 6–8 week‐old male C57BL/6 mice as previously described.^[^ ^29^, ^51^ ^]^ The mice were anesthetized using sodium pentobarbital and subjected to a laminectomy at the 10th thoracic vertebra under microscopic guidance. SCI was induced by dropping a 5 g rod from a height of 6.5 cm using a precision impactor device (RWD Life Science). After the injury, the surgical sites were immediately closed. Postoperative care included manual bladder expression three times daily until normal bladder function was resumed. The mice were randomly partitioned into five groups. Each group received daily tail‐vein injections for five consecutive days. The injections comprised included treatments with different preparations: EVs, Rg1‐EVs, shNC‐Rg1‐EVs, shMYCBP2‐Rg1‐EVs (containing 100 µg of total protein from extracellular vesicles in 100 µL of PBS), or PBS alone as a control (100 µL).

### Evaluation of Behavioral Responses—BMS for Hindlimb Recovery

Hindlimb movement recovery was evaluated using the BMS scores, which assesses trunk posture, limb joint activity, and stability, coordination between the fore and hind limbs, toe clearance during movement, paw placement accuracy, and tail positioning.

### Evaluation of Behavioral Responses—Gait Analysis Via Footprints

At 28 days post‐SCI, footprint analysis was utilized to assess gait and motor coordination. Forepaws and hind paws were dyed blue and red, respectively. Mice were then prompted to walk along a paper‐lined runway to capture and analyze their footprints. Ipsilateral hindlimb step lengths and contralateral hindlimb step widths were measured to evaluate locomotor performance. A longer ipsilateral hindlimb step length was indicative of improved hindlimb locomotion, whereas a smaller contralateral hindlimb step width suggests enhanced stability and coordination.

### Evaluation of Behavioral Responses—Swimming Proficiency Test

The swimming capabilities of mice were tested in a standardized water tank. Mice were trained to swim from one side to the other. According to the Louisville Swimming Scale criteria, evaluation parameters included forelimb reliance, hindlimb movement and alternation, trunk stability, and overall body posture.

### Evaluation of Behavioral Responses—Rotarod Performance Test

Motor skills and coordination were assessed using the rotarod test.^[^ ^52^ ^]^ The latency to fall from an accelerating rotarod was recorded over two trials, and the mean latency was determined.

### Evaluation of Behavioral Responses—VFF Test

Tactility sensitivity was assessed using the von Frey mechanical prick device, where the thickness of the nylon wire and its extension indicated the amount of stimulus provided. Mice were placed on a metal mesh to reach the plantar surface of the paw. The pinprick force was gradually increased from low to high until hindlimb flinching occurred (as a positive response), and the tactile threshold was determined.

### Dihydroethidium (DHE) Staining

Fresh and cryopreserved spinal cord sections were subjected to a 30 min incubation at 37 °C with 2 µmol L^−1^ DHE (Thermo Fisher Scientific), a specific fluorescent dye for superoxide anion detection. The fluorescence emitted by the dye was captured and quantitatively analyzed using a fluorescence microscope.

### TUNEL Staining

On day 28 following SCI, TUNEL staining was performed on spinal cord sections to detect DNA fragmentation indicative of apoptosis as previously described. The sections were permeabilized with 0.3% Triton X‐100 in PBS for 15 min, followed by a 1‐h incubation at ambient temperature in TUNEL staining solution (Beyotime). The stained sections were then examined under a microscope to identify and quantify apoptotic cells.

### Enzyme‐Linked Immunosorbent Assay (ELISA)

Cytokine levels, including TNF‐α, IL‐1β, IL‐6, TGF‐β, IL‐4, and IL‐10, were quantified using commercial ELISA kits following the manufacturer's specified protocols. Optical density was measured at 550 nm and corrected to a standard wavelength of 450 nm using a microplate reader.

### Statistical Analysis

Data analysis was performed using GraphPad Prism 8.0 software (GraphPad Software Inc., USA). Differences between two groups were evaluated using the Student's t‐test, while one‐way or two‐way analysis of variance (ANOVA) was utilized for data analysis involving multiple groups or factors. Results were summarized as mean ± standard deviation. Statistical significance was determined at *p* value < 0.05, with levels of significance denoted as ^*^ *p* < 0.05, ^**^ *p* < 0.01, and ^***^ *p* < 0.001.

### Ethics Approval

All experimental procedures were conducted in accordance with institutional guidelines for the care and use of laboratory animals and protocols, which were approved by the Animal Care and Use Committee of the First Affiliated Hospital of the University of Science and Technology, China (Granted Number: 2024‐N(A)−17).

## Conflict of Interest

The authors declare no conflicts of interest.

## Author Contributions

Y.R., J.W., and T.H. contributed equally to this work. Y.R. performed conceptualization, and data curation, acquired funding, and wrote the original draft. J.W. performed formal analysis and methodology. T.H. performed data curation. Z.S. and C.L. performed investigation and validation. W.L. and W.C. performed methodology and wrote, reviewed, and edited the final manuscript. Y.S. and F.Z. performed conceptualization and project administration. W.Z. performed project administration, acquired funding, and supervised the study.

## Supporting information

Supporting Information

## Data Availability

The data that support the findings of this study are available from the corresponding author upon reasonable request.
