# Supplementary material for: Ginsenoside Rg1 Regulates Immune Microenvironment and Neurological Recovery After Spinal Cord Injury Through MYCBP2 Delivery via Neuronal Cell‐Derived Extracellular Vesicles
Source: Adv Sci (Weinh). 2024 Jun 19;11(31):2402114. doi: 10.1002/advs.202402114 (PMC11336912; doi:10.1002/advs.202402114)
Supplement: Supplementary file 1 — Supporting Information [file ADVS-11-2402114-s001.docx]

Supplementary Figures for

**Ginsenoside Rg1 Regulates Immune Microenvironment and Neurological Recovery after Spinal Cord Injury Through MYCBP2 Delivery via Neuronal Cell-Derived Extracellular Vesicles**

Yuluo Rong^#,^*^,1,2^, Jiaxing Wang^#,3^, Tao Hu^#,1^, Zhongming Shi^1^,Chuandong Lang^1^, Wei Liu^4^, Weihua Cai^3^, Yongjin Sun*^,1^, Feng Zhang*^,1^, Wenzhi Zhang*^,1^

^1^Department of orthopaedics, Centre for Leading Medicine and Advanced Technologies of IHM, The First Affiliated Hospital of USTC, Division of Life Sciences and Medicine, University of Science and Technology of China, Hefei, Anhui, 230001, China.

^2^National Center for Translational Medicine (Shanghai) SHU Branch, Shanghai University, Shanghai, 200444, China

^3^Department of Orthopedics, The First Affiliated Hospital of Nanjing Medical University, Nanjing 210029, Jiangsu, China

^4^Department of Orthopedics, Second Affiliated Hospital of Naval Medical University, Shanghai, 200003, China.

^#^These authors contributed equally to this work

*Correspondence to: Wenzhi Zhang, e-mail: wenzhizhang@ustc.edu.cn; Yuluo Rong, e-mail: rongylspine@ustc.edu.cn; Feng Zhang, e-mail: zfspine@ustc.edu.cn; Yongjin Sun, e-mail: sunyongjin@ustc.edu.cn

**Supplementary Figures**

**
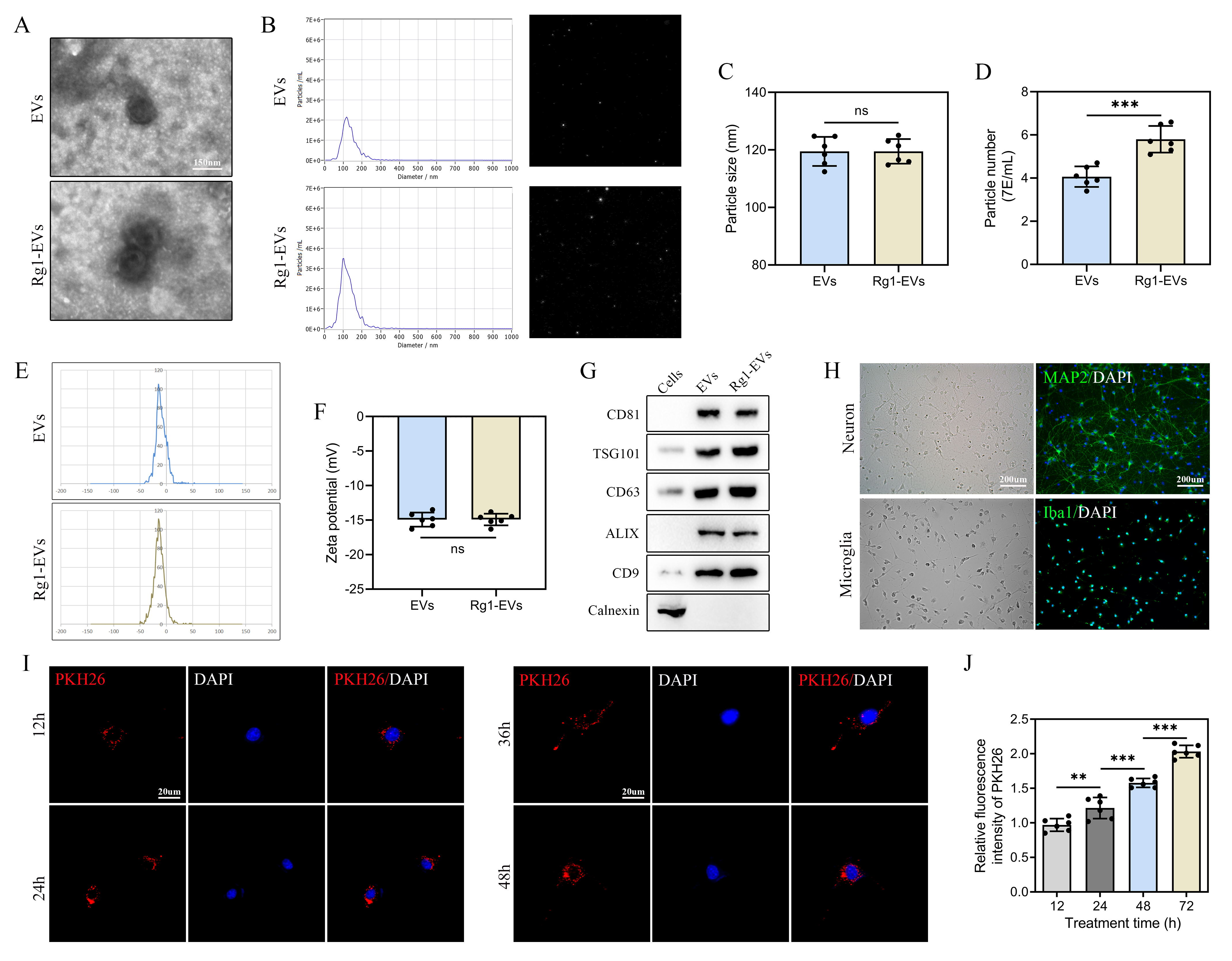
**

**Figure S1: Rg1-EVs identification and uptake.** A. Morphology of EVs and Rg1-EVs under TEM (n=6); B. Particle size analysis of extracellular vesicles in the two groups detected by NTA (n=6); C. Particle size analysis of extracellular vesicles in the two groups (n=6); D. Number of particles per milliliter of extracellular vesicles in the two groups (n=6); E–F. Zeta potential analysis of extracellular vesicles in the two groups (n=6); G. Detection of extracellular vesicle markers by Western blot (n=3); H. Neuronal and microglial cells identified by bright-field maps and immunofluorescence staining; I–J. Analysis of the uptake efficiency of extracellular vesicles by microglia at different co-culture time periods (n=6); **P < 0.01; ***P < 0.001.

**
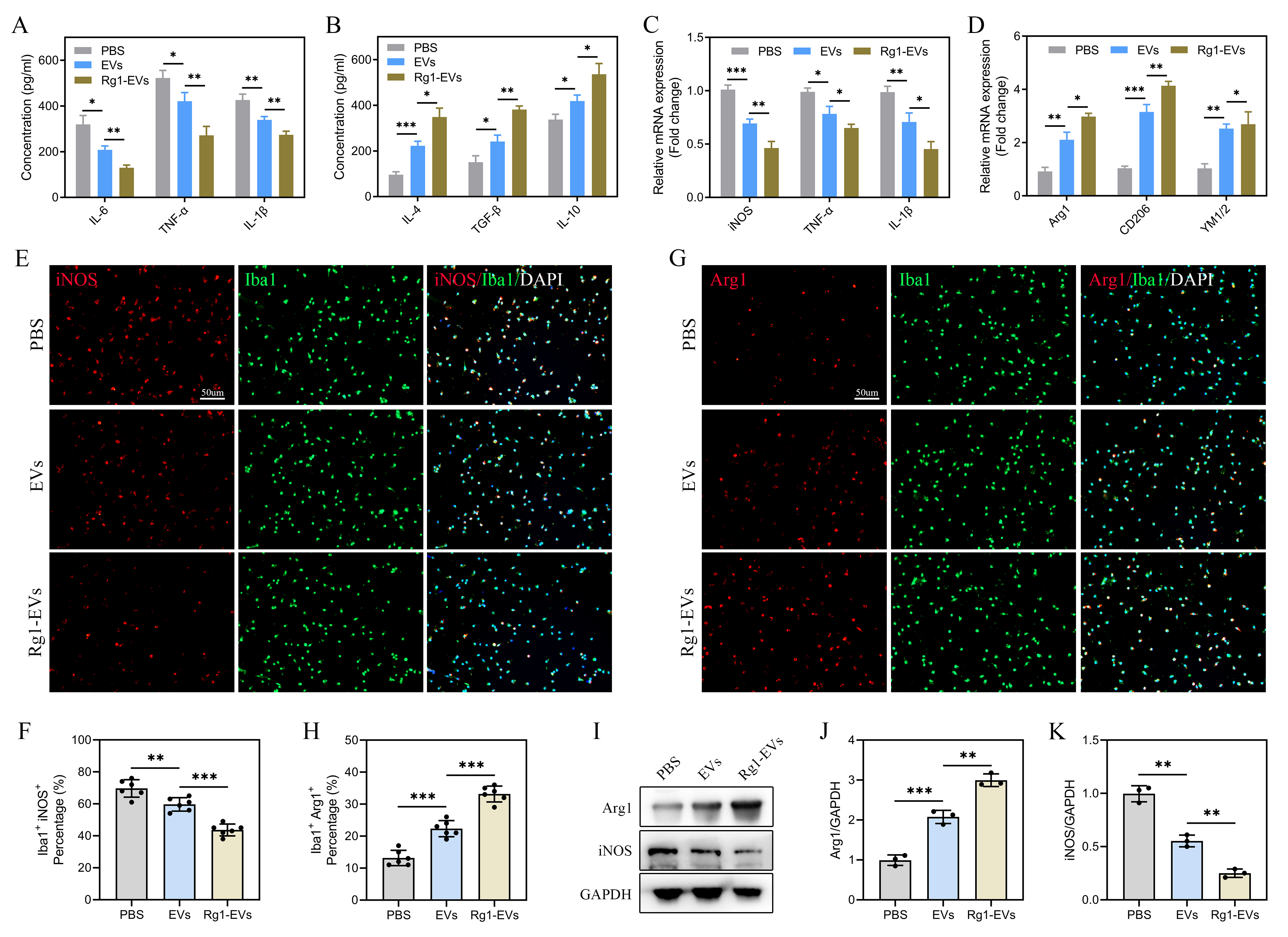
Figure S2: Rg1-EVs reverse the LPS-induced polarization state of microglia.** LPS was added to cell cultures for 24 hours, and PBS, EVs, or Rg1-EVs was added after changing the medium. A–B. ELISA detection of pro- and anti-inflammatory cytokines in the PBS, EVs, and Rg1-EVs groups (n=3); C–D. Detection of the mRNA expression levels of M1- and M2-associated genes by qRT–PCR (n=3); E–F. Immunofluorescence of Iba1 and iNOS in microglia from the three groups and statistical analysis (n=6); G–H. Immunofluorescence staining of Iba1 and Arg1 in microglia from the three groups and statistical analysis (n=6); I–K. Expression of proteins encoded by M1- and M2-related genes detected by Western blot and statistical analysis (n=3); *P < 0.05; **P < 0.01; ***P < 0.001.


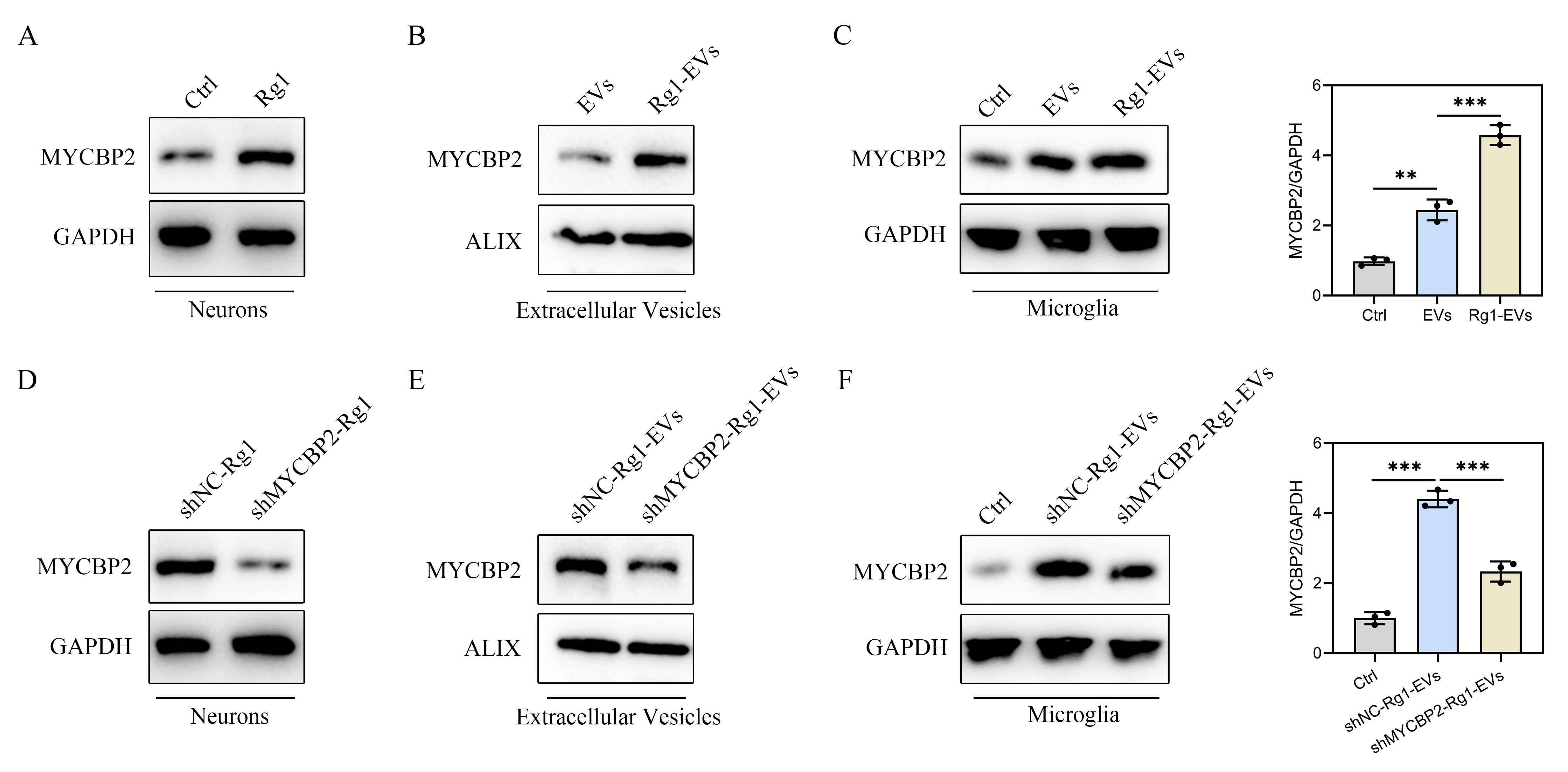


**Figure S3: MYCBP2 is up-regulated in Rg1-pretreated neuronal cells and can be transferred to microglia via extracellular vesicles.** A. Protein expression levels of MYCBP2 in control and Rg1-pretreated neuronal cells were detected by Western blot (n=3); B. Protein expression levels of MYCBP2 in EVs and Rg1-EVs were detected by Western blot (n=3); C. Protein expression levels of MYCBP2 in microglia were detected by Western blot and statistical analysis (n=3); D. Knockdown efficiency of shMYCBP2 in Rg1-pretreated neuronal cells (n=3); E. MYCBP2 protein expression levels in shNC-Rg1-EVs and shMYCBP2-Rg1-EVs were detected by Western blot (n=3); F. MYCBP2 protein expression levels in microglia were detected by Western blot and statistical analysis (n=3); **P < 0.01; ***P < 0.001.


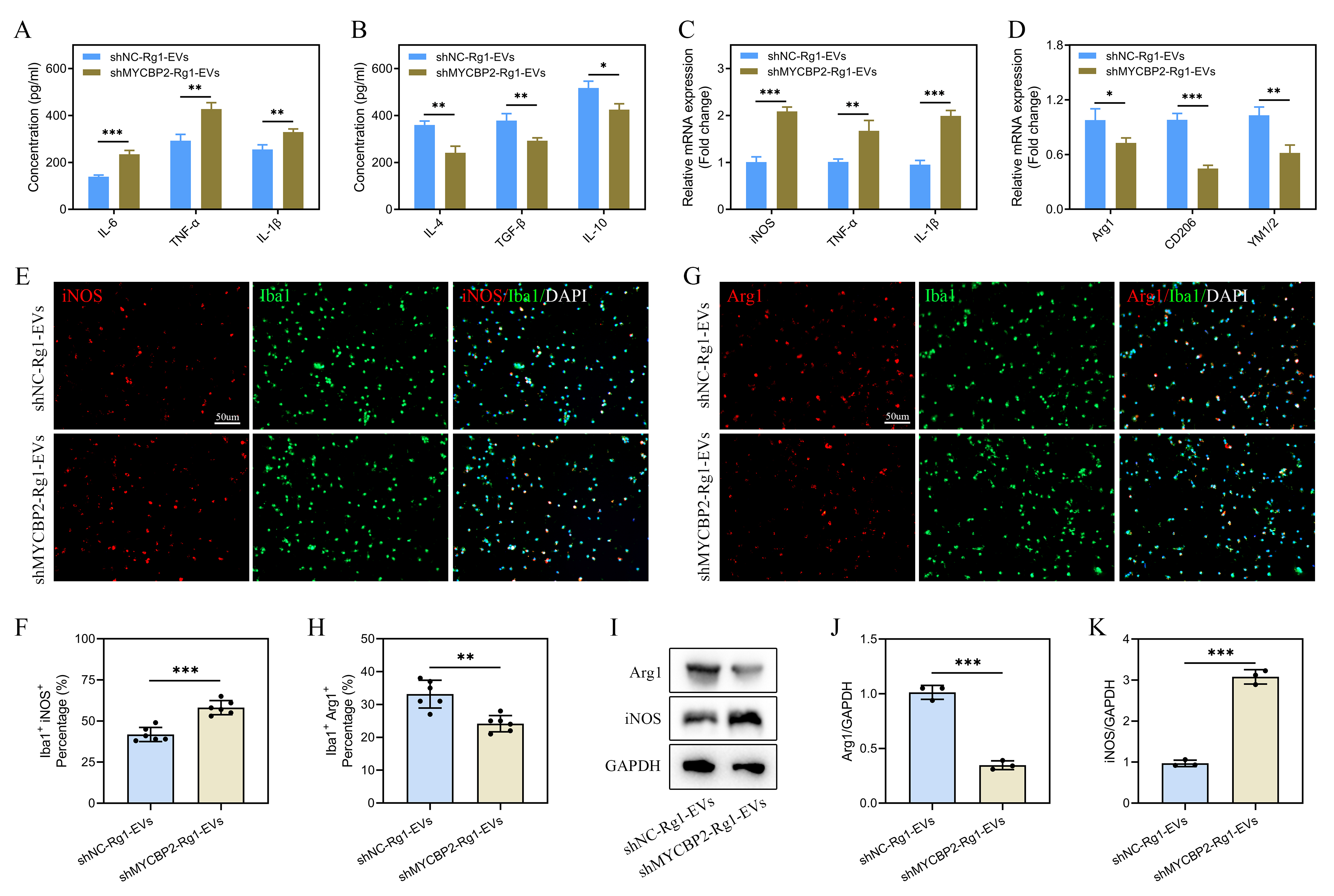


**Figure S4: Rg1-EVs convert microglia to the M2-type polarization state by delivering MYCBP2 in vitro.** A–B. ELISA for pro- and anti-inflammatory cytokines in the shNC-Rg1-EVs group and the shMYCBP2-Rg1-EVs group (n=3); C–D. Detection of the mRNA expression levels of M1- and M2-related genes by qRT–PCR (n=3); E–F. Immunofluorescence staining of Iba1 and iNOS in microglia from both groups and statistical analysis (n=6); G–H. Immunofluorescence staining of Iba1 and Arg1 in microglia from both groups and statistical analysis (n=6); I–K. Detection of proteins encoded by M1- and M2-related genes by Western blot and statistical analysis (n=3); *P < 0.05; **P < 0.01; ***P < 0.001.


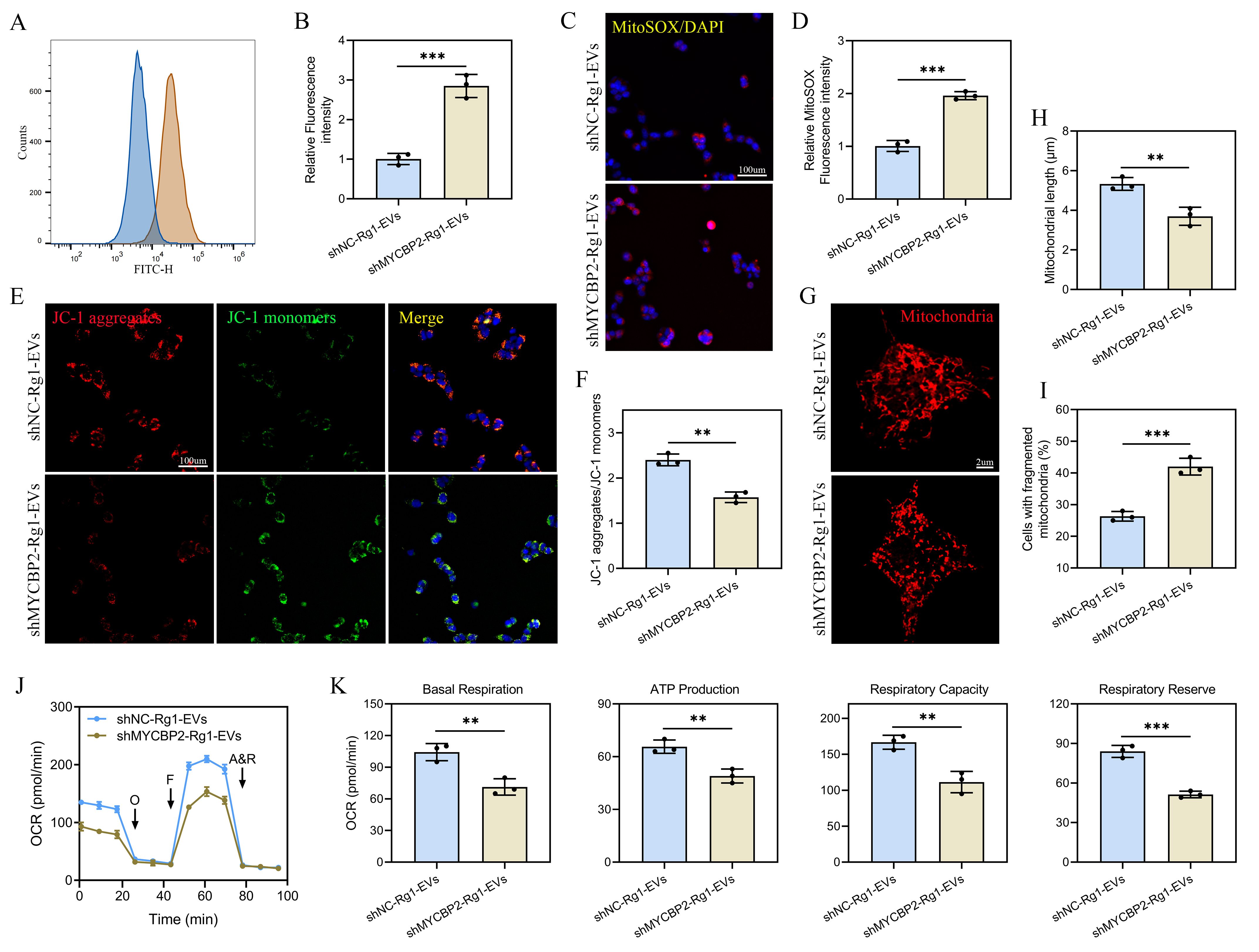


**Figure S5: Rg1-EVs downregulate ROS and regulate mitochondrial function by delivering MYCBP2 in vitro.** A–B. Flow cytometry detection of ROS in microglia in the shNC-Rg1-EVs group and the shMYCBP2-Rg1-EVs group and statistical analyses (n=3); C–D. Immunofluorescence staining and quantitative analyses of MitoSOX in both groups (n=3); E–F. Determination and quantification of mitochondrial potential by JC-1 aggregates/JC-1 monomers in both groups (n=3); G–I. Representative images of mitochondrial morphology, quantification of mitochondrial length, and percentage of cells with mitochondrial debris in both groups (n=3); J. Detection of OCR in microglia from both groups using the Seahorse Bioscience XFp analyzer (n=3); K. Determination of mitochondrial activity, including basal respiration, ATP production, respiratory capacity, and respiratory reserve, in both groups (n=3); **P < 0.01; ***P < 0.001.
